# Transcriptome-Wide Identification and Characterization of the *JAZ* Gene Family in *Mentha canadensis* L.

**DOI:** 10.3390/ijms22168859

**Published:** 2021-08-17

**Authors:** Dong-Bei Xu, Ya-Nan Ma, Teng-Fei Qin, Wei-Lin Tang, Xi-Wu Qi, Xia Wang, Rui-Cen Liu, Hai-Ling Fang, Ze-Qun Chen, Cheng-Yuan Liang, Wei Wu

**Affiliations:** 1 College of Agronomy, Sichuan Agricultural University, Chengdu 611130, China; weilintang2021@163.com (W.-L.T.); wangxiayuyao@hotmail.com (X.W.); liuruicen@126.com (R.-C.L.); 2 Institute of Botany, Jiangsu Province and Chinese Academy of Sciences (Nanjing Botanical Garden Mem. Sun Yat-Sen), No. 1 Qianhu Houcun, Zhongshanmen Wai, Nanjing 210014, China; mayanan613@gmail.com (Y.-N.M.); xiwuqi@cnbg.net (X.-W.Q.); fanghailing2013@163.com (H.-L.F.); zequnchen@126.com (Z.-Q.C.); 3 Henan Collaborative Innovation Center of Modern Biological Breeding, Henan Institute of Sciences and Technology, Xinxiang 453003, China; qintengfeisam@163.com

**Keywords:** *Mentha canadensis* L., jasmonate, McJAZ protein, phylogenetic analysis, gene expression, protein-protein interaction

## Abstract

Jasmonate ZIM-domain (JAZ) proteins are the crucial transcriptional repressors in the jasmonic acid (JA) signaling process, and they play pervasive roles in plant development, defense, and plant specialized metabolism. Although numerous *JAZ* gene families have been discovered across several plants, our knowledge about the *JAZ* gene family remains limited in the economically and medicinally important Chinese herb *Mentha canadensis* L. Here, seven non-redundant *JAZ* genes named *McJAZ1*–*McJAZ7* were identified from our reported *M. canadensis* transcriptome data. Structural, amino acid composition, and phylogenetic analysis showed that seven McJAZ proteins contained the typical zinc-finger inflorescence meristem (ZIM) domain and JA-associated (Jas) domain as conserved as those in other plants, and they were clustered into four groups (A-D) and distributed into five subgroups (A1, A2, B1, B2, and D). Quantitative real-time PCR (qRT-PCR) analysis showed that seven *McJAZ* genes displayed differential expression patterns in *M. canadensis* tissues, and preferentially expressed in flowers. Furthermore, the *McJAZ* genes expression was differentially induced after Methyl jasmonate (MeJA) treatment, and their transcripts were variable and up- or down-regulated under abscisic acid (ABA), drought, and salt treatments. Subcellular localization analysis revealed that McJAZ proteins are localized in the nucleus or cytoplasm. Yeast two-hybrid (Y2H) assays demonstrated that McJAZ1-5 interacted with McCOI1a, a homolog of *Arabidopsis* JA receptor AtCOI1, in a coronatine-dependent manner, and most of McJAZ proteins could also form homo- or heterodimers. This present study provides valuable basis for functional analysis and exploitation of the potential candidate *McJAZ* genes for developing efficient strategies for genetic improvement of *M. canadensis*.

## 1. Introduction

Jasmonates (JAs), are lipid-derived natural phytohormones that act as ubiquitous moderators of a broad range of biological processes including plant growth [1,2,3,4,5,6,7], defense against pathogen infection, herbivorous insect attack, and abiotic stress [8,9,10,11]. JAs are also active in specialized metabolism through programing the transcripts of JA-responsive genes across the plant kingdom, from gymnosperms to angiosperms [12,13,14,15,16,17], implying that the usage of JAs or JA-associated networks may be a valuable strategy for future plant improvement.

JA biosynthesis has been deeply explored in dicotyledonous and monocotyledonous plants such as *Arabidopsis* and rice [5]. These results indicate that JA biosynthesis is initiated by a series of sequential oxygenations of α-linolenic acid using lipoxygenase (LOX), allene oxide synthase (AOS), and allene oxide cyclase (AOC), leading to the synthesis of 12-oxo-phytodienoic acid (OPDA). OPDA is then catalyzed and converted to (+)-7-iso-JA by an OPDA reductase 3 (OPR3) and three rounds of β-oxidation; thus, the above de novo JA biosynthesis is well known as the octadecanoid pathway [18]. Afterwards, the studies found that (+)-7-iso-Jasmonoyl-L-isoleucine (JA-Ile), synthesized from (+)-7-iso-JA by jasmonate amino acid synthetase 1 (JAR1), is considered the major form of active JA in plants [18,19].

In addition, substantial research has found that JA perception and signal transduction, as well as transcript reprogramming of downstream genes, are a delicate and dynamic process containing numerous regulators [20,21]. In *Arabidopsis*, under normal conditions with a lower JA level, a series of JASMONATE ZINC-FINGER INFLORESCENCE MERISTEM (ZIM)-DOMAIN (JAZ) proteins act as transcriptional repressors of JA signaling. JAZ function to recruit TOPLESS (TPL) or TPL-related proteins (TPRs) by interacting with the adaptor protein, Novel Interactor of JAZ (NINJA), which together form the co-repressor complex which represses the transcriptional regulatory activity of transcription factors (TFs) [22]. This regulatory process suppresses the expression of JA-responsive genes and attenuates various downstream JA-mediated responses. In contrast, the activity of JAZ proteins is delicately controlled by the JA receptor, CORONATINE INSENSITIVE 1 (COI1) [23], an F-box protein which is a component of the Skp1-Cullin-F-box (SCF) E3 ubiquitin ligase complex (SCF^COI1^). Under stress stimuli, JA and Ile conjugate to form the bioactive JA-Ile, which is then recognized by COI1, forming the COI1-Ile complex, which interacts with JAZ repressors. This interaction then triggers the ubiquitination and subsequent degradation of the JAZ repressors by the action of the SCF^COI1^ complex and the 26S proteasome [19], which releases the TFs to regulate the transcripts of diverse JA-responsive genes involved in JA-regulated biological processes [4,15,24]. Subsequently, numerous studies have uncovered that the multifaceted function of JA is highly attributable to the pairwise interactions of the different TFs and JAZ repressors which regulate the transcription of target genes in a temporal and spatial-dependent manner in plants [4,8,14,25,26]. In addition, it was reported that JA has synergistic or antagonistic functions with abscisic acid (ABA), gibberellic acid (GA), ethylene (ETH), and salicylic acid (SA) in controlling plant growth and stress responses. Notably, JAZ proteins often act as an important hub for regulating the crosstalk of JA and other hormones to dynamically adjust growth, and accurately cope with environmental stresses [27,28,29,30]. Thus, JAZ repressors are not only a crucial link in the COI1-JAZ-TFs module that regulates paths of JA signal transduction, but also a major regulator that integrates multiple signaling pathways.

The JAZ protein family is known as a member of the plant-specific TIFY superfamily, which contains TIFY, ZIM-like (ZML), PEAPOD (PPD), and JAZ protein subfamilies [31]. Of these, JAZ proteins have a conserved TIFY (also known as ZIM) domain containing a core TIF[F/Y]XG motif near the N-terminal region of JAZ protein sequences, and a JA-associated (also named CCT_2) domain which contains the extremely conserved sequence “SLX_2_FX_2_KRX_2_RX_5_PY”, located in the C-terminus [31,32,33]. In fact, the TIFY domain is not only necessary for JAZ proteins to recruit transcriptional co-repressors like TPL or TPRs via interacting with the NINJA protein [22], but also functions to mediate the paired interactions of JAZ proteins to form homo- and heterodimers [34]. The Jas domain, on the other hand, mediates the JA-Ile dependent interaction of JAZ protein with COI1, or the interaction of JAZ proteins with various TFs [30]. Interestingly, several JAZ proteins, like *Arabidopsis* AtJAZ5-AtJAZ8 and AtJAZ13 also have additional ethylene-responsive elements binding factor-associated amphiphilic repression (EAR) motifs that can recruit TPL/TPRs in the absence of NINJA [35,36], thus providing additional functional paths for JAZ proteins in plants.

JAZ proteins were first identified in *Arabidopsis thaliana* in 2007 [19], and are widely present in the green plant lineage. For instance, there are 13, 34, 15, 18 and 13 JAZ proteins identified in *A. thaliana* [33], *Triticum aestivum* [37], *Oryza sativa* [38], *Hevea brasiliensis* [39], and *Solanum lycopersicum* [40]. Next, functional studies indicated that different *JAZ* genes have varied biological function. *Arabidopsis* AtJAZ4 is involved in regulating the development of root, hypocotyl, and petiole [41], and AtJAZ proteins promote growth and reproductive success partially by attenuating the abnormal metabolic effects of an unrestrained immune response [42]. Likewise, MpJAZ, a homolog of AtJAZ, regulated cell growth and reproductive fitness in *Marchantia polymorpha* [43]. In addition, overexpression of *VqJAZ7* enhanced resistance to powdery mildew by controlling cell death and the accumulation of superoxide anions [44]. TaJAZ1 positively regulated powdery mildew resistance via up-regulating expression of defense-related gene *TaPR1/2* and increasing reactive oxygen species accumulation in wheat [45]. In rice, up- or down-regulation of *OsJAZ1* or *OsJAZ9* resulted in drought response or salt sensitivity [46,47]. It has also been reported that *Glycine soja* GsJAZ2 positively regulate salt and alkali stress tolerance [48]. More importantly, several lines of evidence have revealed the key role of JAZ proteins in biosynthesis of plant specialized metabolism such as alkaloids, artemisinin, and tanshinone. In *Artemisia annua*, AaJAZ8 negatively regulated the biosynthesis of artemisinin, a first line of defense against malaria, in response to JA elicitation [24]. In *Salvia miltiorrhiza*, overexpression of *SmJAZ3* and *SmJAZ9* reduced tanshinone content [49]. Therefore, *JAZ* genes have a broad role in regulating adaptability to environmental challenges, and modulate development and specialized metabolism in different plants.

*Mentha canadensis* L. belongs to the *Lamiaceae* family, is a widely cultivated aromatic plant, and is also a common medicinal plant in China [50,51]. Furthermore, *M. canadensis* and its relatives are a source of high-value natural products including essential oils (EOs) widely used in cosmetics, aromatherapy industries, and also exhibit natural antioxidant properties, antibacterial properties, and pharmaceutical value [52,53,54]. These species are also promising bioenergy feedstocks for ethanol production [50,55] or use as natural insecticides for pest management in agriculture [56]. Meanwhile, pioneering studies have found that monoterpenes including (−)-Limonene, (−)-Carvone, (+)-Menthofuran, (−)-Menthone, and (−)-Menthol, a kind of C_10_ class of terpenoid, are the major constituents of EOs in mint. The de novo biosynthetic pathway of monoterpenoid EOs derived from the upstream methylerythritol phosphate (MEP) pathway has been systematically discovered in the two reported mint cultivars peppermint (*Mentha × piperita* L.) and spearmint (*Mentha spicata* L.) in a context-dependent manner. Meanwhile, research into the physiology and molecular biology of these compounds uncovered that the yield and composition of EOs is varied among the mint family in response to SA [57], JA [58], GA [59], high temperatures [53], salinity and copper stress [60], and this phenomenon may be associated with transcriptional regulation and epigenetic modification of EO biosynthetic enzyme genes, and the catalytic properties of a series of EO biosynthetic enzymes [61,62,63].

By contrast, previous genetic studies of *M. canadensis* are scant due to a lack of sequence data and mainly focused on chemical composition and medicinal usage [64], plant growth and physiological assays [65], and the functional analysis of putative EO biosynthetic enzyme genes including the Geranyl diphosphate synthase subunit (GPPS), and Limonene synthase (LS) [66]. However, little is known about gene families that are closely associated with EO biosynthesis in *M. canadensis*. Recently, transcriptome sequencing of *M. canadensis* under Methyl jasmonate (MeJA) treatment uncovered that JA was involved in promoting EO biosynthesis [67], similar to earlier reports [58], and the release of transcriptome data of *M. canadensis* provides basic information to analyze the key genes involved in regulating different aspects of *M. canadensis* including EO biosynthesis. As mentioned, JAZ proteins play an important role in JA signaling and JA-mediated secondary metabolism. Thus, based on the reported transcriptome sequencing data of *M. canadensis*, we first identified *McJAZ* family genes, analyzed their sequence properties, structural features, phylogenetic relationships, amino acid composition, and expression profiles in different tissues or under JA, ABA, and abiotic stress treatments. In addition, our results showed that McJAZ proteins were localized to the nucleus or cytoplasm, and most of them interacted with McCOI1a in a coronatine-dependent manner, and also formed homo- or heterodimers in *M. canadensis*. Thus, the current results enrich our knowledge of *JAZ* family genes and provide a valuable foundation for functional analysis and exploitation of the candidate *McJAZ* genes for genetic improvement of *M. canadensis*.

## 2. Results

### 2.1. Identification and Molecular Cloning of McJAZ Genes from M. canadensis

**Figure 1:**
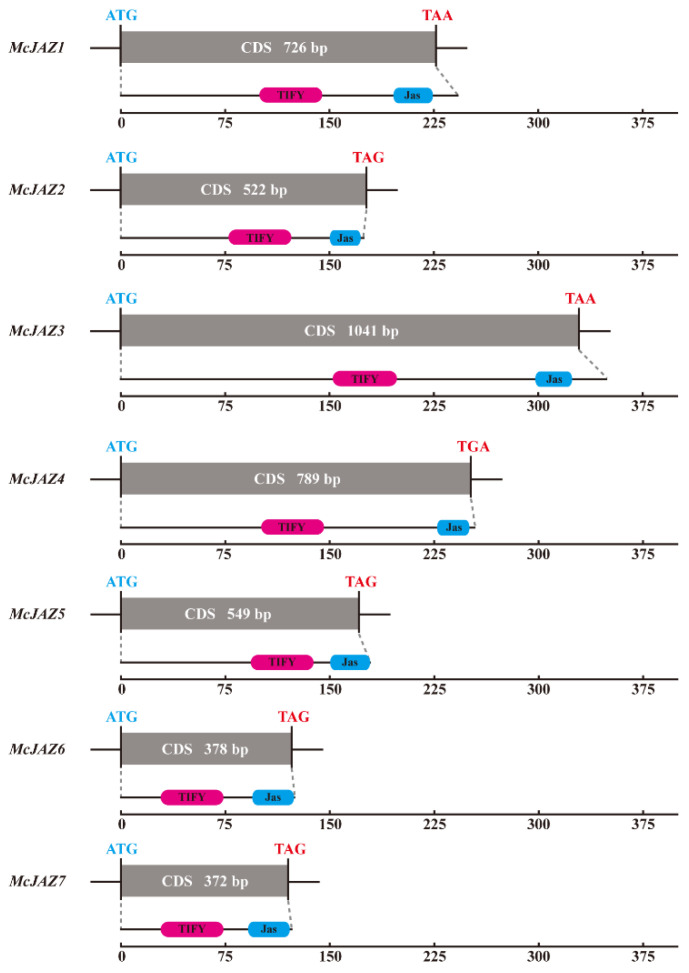
***McJAZ* genes and domain architectures of McJAZ proteins in *M. canadensis*.** Shown above are the coding sequences (CDs) of the seven focal *McJAZ* genes, and the domain architectures of these McJAZ proteins. The McJAZ proteins share a conserved TIFY domain (indicated by a pink box), and Jas domain (indicated by a blue box) with other JAZ homologous proteins in other plant species.

To identify the putative *McJAZ* family genes in *M. canadensis*, in total, twelve well-known JAZ protein sequences from *A. thaliana* [19] and nine reported JAZ protein sequences from *A. annua* [24] were collected and used as queries to perform a BLASTP search based on the previous transcriptome data (SRP132644) at the National Center for Biotechnology Information (NCBI) [67]. Finally, we identified and annotated eight non-redundant full-length *McJAZ* genes in the *M. canadensis* transcriptome database. Next, we obtained seven amplicons corresponding to seven distinct full-length *McJAZ* genes through PCR amplification with gene specific primers. Subsequently, the purified amplicons were ligated with pClone007 Blunt Simple vector, and the resulting products were transformed into *Escherichia coli* DH5α and sequenced, respectively. After this, we successfully cloned seven full-length *McJAZ* genes, and denoted them as *McJAZ1*-*McJAZ7* (Figure 1 and Table 1). As expected, all of these McJAZ proteins contained a N-terminus conserved TIFY domain and a C-terminus Jas domain (also named CCT_2 motif). These findings were further corroborated by using the online SMART tools (http://smart.embl-heidelberg.de/, accessed on 25 April 2021) with the default parameters (Figure 1 and Table 1).

Next, sequence analysis showed that the length of the *McJAZ* genes coding sequences (CDs) ranged from 372 (*McJAZ7*) to 1041 (*McJAZ3*) base pairs (bp), which was similar in length to the known *AtJAZ* CDs ranging from 396 (*AtJAZ8*) to 1059 (*AtJAZ3*) bp in *A. thaliana* [19]. Furthermore, these candidate *McJAZ* genes encoded predicted products which varied in length from 123 (McJAZ7) to 346 (McJAZ3) amino acids (aa) residues, with molecular weights (*M*_W_) ranging from 13.87 kilodalton (kDa) (McJAZ7) to 36.89 kDa (McJAZ3), and the isoelectric point (pI) ranged from 6.12 (McJAZ2) to 9.96 (McJAZ6) (Table 1). Notably, the pI features of most of McJAZ proteins except for McJAZ2 were greater than 7, indicating that most of the McJAZ proteins were basic proteins. Taken together, all of these results provide an earlier basis for further characterization of JAZ family proteins or related networks in *M. canadensis*.

### 2.2. Sequence Alignment and Phylogenetic Analysis of McJAZ Genes

To understand the evolutionary and phylogenetic relationships of the *JAZ* family genes between *M. canadensis* and other known species including *A. thaliana*, *O. sativa*, and *A. annua*, a phylogenetic tree was constructed based on an unrooted neighbor-joining (NJ) method using twelve AtJAZ proteins from *A. thaliana*, fifteen OsJAZ proteins from *O. sativa*, nine AaJAZ proteins from *A. annua*, and seven McJAZ proteins from *M. canadensis*. The results showed that all the JAZ proteins were clustered into four groups (Groups A–D) and further classified into A1, A2, B1–B3, C, and D subgroups according to the reported phylogenetic analysis of AtJAZ proteins in *Arabidopsis* [34]. Among them, all McJAZ proteins were distributed into 5 subgroups, such that subgroup A1 contains McJAZ5, subgroup A2 contains McJAZ3 and McJAZ4, subgroups B1 and B2 contain McJAZ1 and McJAZ2 respectively, and McJAZ6, McJAZ7 belongs to subgroup D (Figure 3).

**Figure 2:**
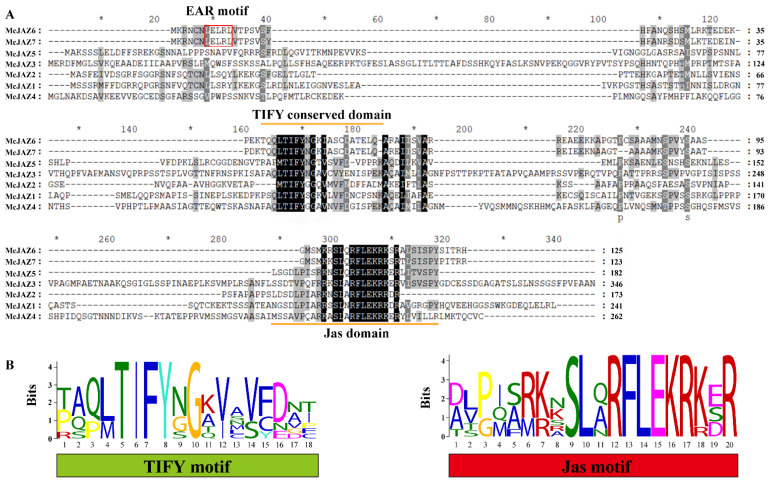
**Multiple sequence alignment of McJAZ proteins from *M. canadensis*.** (**A**) Alignment of the amino acid sequences of McJAZ proteins from *M. canadensis*. The EAR motif is indicated by a red rectangle. The conserved TIFY and Jas domains are outlined with a saffron yellow straight line. (**B**) The sequence logos of the TIFY and Jas motif are highly conserved across all McJAZ proteins. The height of each stack indicates conservation of the sequence at the labeled positions, and the height of each letter within each stack indicates the relative frequency of the corresponding amino acid.

To analyze the similarity of the homologous sequences among the candidate McJAZ proteins, multiple sequence alignment analysis of the seven McJAZ proteins or the pairwise McJAZ proteins was carried out. The results showed that except for McJAZ6 and McJAZ7, which shared 80.80% identity at the amino acid sequence level (Supplemental Table S1), the rest of pairwise McJAZ proteins contained differential amino acid identity ranging from 6.94% to 27.80% shared identity, analyzed by local DNAMAN 6.0 software (Figure 2A and Supplemental Table S1). In contrast to the sequences overall, the typical TIFY and Jas domains were conserved in all McJAZ proteins in *M. canadensis* (Figure 2A). Similarly, the yield sequence logos drawn using the MEME tool also showed highly conserved amino acid sequence within the TIFY and Jas domains (Figure 2B). In addition, we also found that McJAZ6 and McJAZ7 possessed the typical EAR (LELRL) motif in the N-terminal region that is widely present in JAZ proteins across different plants (Figure 2A). Interestingly, evidence suggests that the TIFY or Jas motif is responsible for mediating protein-protein interactions for itself or other partners [19,34]. Overall, these results showed the possibility that the function of the seven McJAZ proteins may be conserved within *M. canadensis*.

### 2.3. Tissue Expression Patterns of McJAZ Genes

To further investigate expression patterns of *McJAZ* genes in *M. canadensis*, we analyzed the expression levels of each *McJAZ* gene across different tissues (Figure 4A) including roots, stems, young leaves, and flowers using quantitative real-time PCR (qRT-PCR) assays. The results showed that all *McJAZ* genes were broadly expressed in all evaluated tissues and displayed the highest expression in flowers (Figure 4). More specifically, expression patterns fluctuated across tissues and were significantly different for each *McJAZ* gene and could be classified into three types. As shown in Figure 4B, the expression profiles of *McJAZ1*, *McJAZ2*, *McJAZ4*, *McJAZ5*, and *McJAZ6* showed similar expression patterns, with the highest levels in flowers, followed by young leaves, stems, and relatively low expression in roots compared with other tissues. In addition, *McJAZ3* was found to be most highly expressed in flowers, and its expression was successively reduced in young leaves, roots, and stems. We next found that *McJAZ7* showed highest expression in flowers, moderately expression in stems and young leaves, and low expression in roots (Figure 4B). These results suggest that the seven *McJAZ* genes studied here were constitutively expressed in all four *M. canadensis* tissues, and most of them were highly abundant in the flower and young leaf tissues.

### 2.4. Expression Profiles of McJAZ Genes in Response to JA and ABA Treatments

Numerous reports have shown that MeJA and ABA play a vital role in regulating plant development, stress response, and various physiological processes through controlling the expression patterns of downstream genes [8,18,68,69]. Hence, to next investigate the potential roles of *McJAZ* genes in response to MeJA and ABA, qRT-PCR was performed to analyze the relative expression profiles of these *McJAZ* genes in *M. canadensis* that had been treated with MeJA or ABA. Based on qRT-PCR results, we found that the expression pattern of each *McJAZ* gene was variable under MeJA or ABA treatment (Figure 5). Under MeJA treatment, the transcript levels of seven *McJAZ* genes were distinctly up-regulated (Figure 5A), similar to previously reported transcriptome data (Supplemental Figure S1) [67]. In general, *McJAZ1*, *McJAZ2*, *McJAZ6*, and *McJAZ7* transcript levels displayed a rapid increase, peaking at 1 h after MeJA exposure, and then gradually decreased at 3, 6, and 12 h, and returned to approximately control levels (CK 0 h) after prolonged MeJA exposure (24 h). In contrast, *McJAZ3*, *McJAZ4*, and *McJAZ5* expression increased at 1 h after MeJA exposure, and significantly increased until a peak after 3 h, then gradually decreased after prolonged MeJA exposure (6, 12, and 24 h) (Figure 5A).

In addition, we found that except for *McJAZ4*, the transcripts of *McJAZ* genes were also dynamically affected by exogenous ABA treatment. Among them, *McJAZ2*, *McJAZ5*, and *McJAZ6* transcript levels were significantly up-regulated at 1, 3, and 24 h for *McJAZ2*, at 1, 12, and 24 h for *McJAZ5*, and at 1 and 24 h for *McJAZ6* after ABA exposure (Figure 5B). In particular, the expression levels of *McJAZ2*, *McJAZ5*, and *McJAZ6* reached their highest levels at 14, 21, and 7 times higher than the control (CK 0 h) at 1 h respectively. Additionally, the expression of *McJAZ1* was slightly up-regulated at 1 h, but down-regulated subsequently (3, 6, 12, and 24 h), and reached its lowest level at 6 h after ABA exposure. The transcript level of *McJAZ3* was not obvious different at 1, 3, and 24 h, but was down-regulated at 6 and 12 h, compared with control (CK 0 h). *McJAZ7* expression was slightly decreased after 1, 3, 6, and 12 h of ABA exposure, and then returned to a level comparable to that seen at the control time point (CK 0 h). These results revealed that *McJAZ* genes expression responds to JA and ABA, and may be involved in JA or ABA signaling pathway in *M. canadensis*.

### 2.5. McJAZ Genes Are Involved in Response to Drought and Salt Stress

Abiotic stress is known to severely limit plant growth and development, and affect multiple physiological processes, and the species’ geographical distribution. To further examine the potential functions of *McJAZ* genes in response to abiotic stresses, qRT-PCR assays were employed to investigate the expression patterns of *McJAZ* genes in *M. canadensis* treated with drought and salt stress. The results showed that, under drought stress treatment, the expression levels of the seven *McJAZ* genes were significantly up-regulated at the individual time point, and expression levels varied by gene (Figure 6A). Expression of *McJAZ1*, *McJAZ2*, and *McJAZ7* or *McJAZ3* reached maximum levels that were 2 to 37 times higher than the control at 12 h or 1 h. In addition, *McJAZ4* expression was gradually induced by drought and peaked at 12 h, later declining to a level similar to the control (CK 0 h) at 24 h after drought treatment. On the other hand, the transcripts of *McJAZ5* and *McJAZ6* were also gradually induced, and reached their highest levels at nearly 8-fold and 39-fold at 24 h under drought treatment compared with control (Figure 6A).

In addition, seven *McJAZ* genes had a fluctuating and significantly different transcriptional response to salt treatment (Figure 6B). Among them, *McJAZ1* and *McJAZ4* expression were down-regulated by salt treatment at a subset of time points compared with control (CK 0 h) (Figure 6B). *McJAZ3* expression was generally down-regulated to varying degrees at each time point after salt treatment, and reached the lowest levels at 24 h. Expression analysis of *McJAZ* genes also showed that *McJAZ2* and *McJAZ7* expression were up-regulated at 1–12 h, and reached maximum levels of more than 6-fold or 4-fold compared with control (CK 0 h) at 12 h or 6 h, respectively. In addition, the transcript levels of *McJAZ5* and *McJAZ6* were up-regulated, and peaked at more than 7-fold or 9-fold compared with the control at 3 h, gradually decreased after prolonged salt treatment (6 and 12 h), then returned to levels similar to control (CK 0 h) at 24 h after salt treatment. These results implied that most of the *McJAZ* genes were sensitive to drought and salt stress treatments, and are likely involved in drought and salt stress related signaling networks in *M. canadensis*.

### 2.6. Subcellular Localization of McJAZ Proteins

To further determine subcellular localization of McJAZ proteins, subcellular localization was first predicted using an online tool (http://cello.life.nctu.edu.tw/, accessed on 30 April 2021). Preliminary results showed that seven McJAZ proteins were predicted to be targeted to nuclei (Table 1). Accordingly, we next constructed an *McJAZs-GFP* (Green Fluorescence Protein) fusion expression vector under control of the CaMV 35S promoter, and subsequently these fusion proteins or the GFP control were transiently expressed in *Nicotiana benthamiana* leaf cells. The subcellular localization results showed that, in contrast to GFP, which was localized uniformly throughout the cell, most of the McJAZ proteins were exclusively located in the nucleus marked with the red arrow in the leaves of *N. benthamiana* (Figure 7), which is consistent with the previously predicted subcellular localization (Table 1). Meanwhile, McJAZ2 and McJAZ3 were also observed with cytoplasmic localization apart from nuclear (Figure 7). This phenomenon is consistent with recent reports that some JAZ proteins of *Lycoris aurea* including LaJAZ1 show both nuclear and cytosolic localization [70].

### 2.7. McJAZ Proteins Interact with McCOI1a

Previous studies report that JAZ proteins play a vital role in JA response through interacting with the JA receptor, CORONATINE INSENSITIVE 1 (COI1) in a JA-Ile- or coronatine (COR, a functional mimic of bioactive JA-Ile)-dependent manner rather than a JA- or MeJA-dependent manner [19,23,71,72]. Some JAZ proteins in non-model plants may also interact with AtCOI1 [71]. To determine the role of McJAZ proteins in the JA response in *M. canadensis*, we first examined the interaction between AtCOI1 and McJAZ proteins by yeast two-hybrid (Y2H) assays. As shown in Figure 8, all of the yeast transformants grew normally on the control medium (DDO, Figure 8A), whereas none of the McJAZ proteins interacted with AtCOI1 regardless of whether the selection medium (QDO) was supplemented with the JA-Ile mimic, COR (Figure 8B,C). Subsequently, given that protein-protein interaction might occur in a context-dependent manner in different plant species, we next cloned the homologous protein of AtCOI1, designated this as McCOI1a given that other unidentified copies of McCOI1 may exist in *M. canadensis*, and then examined the interaction between McCOI1a and McJAZ proteins. As expected, interaction was also not detected for McCOI1a and all seven McJAZ proteins under selection medium (QDO) in the absence of COR (Figure 8B). In contrast, except for McJAZ6 and McJAZ7, most of McJAZ proteins including McJAZ1, McJAZ2, McJAZ3, McJAZ4 and McJAZ5 interacted with McCOI1a in selection medium (QDO) supplemented with COR respectively (Figure 8C), suggesting that a conserved interaction between COI1 and JAZ is present in different plant species.

### 2.8. Homo- and Heterodimeric Interaction of McJAZ Proteins

Our previous results showed that all of the McJAZ proteins have a conserved TIFY domain (Figure 1 and Figure 2), and previous evidence has also demonstrated that the TIFY domain is involved in regulating homo- and heterodimeric interactions among JAZ proteins [34]. Thus, we further examined the possibility that the seven McJAZ proteins could form homo- or heterodimers by testing 49 combinations among the seven McJAZ proteins in the Y2H assays (Figure 9A,B). As expected, McJAZ proteins could indeed form homo- or heterodimeric pairs as evidenced by growth of yeast in selective medium SD/-L/-T/-H/-A. As shown in Figure 9B, three out of the seven McJAZ proteins including McJAZ2, McJAZ3, and McJAZ5 could form homodimers by reciprocal interaction in yeast. Meanwhile, the different heterodimeric interactions were also observed among the seven McJAZ proteins (Figure 9B). For instance, the interactions between McJAZ1 and McJAZ5, McJAZ2 and McJAZ4, McJAZ5 or McJAZ7, McJAZ4 and McJAZ5 were observed in reciprocal transformations in yeast. Furthermore, McJAZ1 only interacted with McJAZ3, McJAZ4 as the prey, and McJAZ2 interacted with McJAZ3, McJAZ6 as the prey. McJAZ3 could interact with McJAZ1, McJAZ2, McJAZ4 and McJAZ5 as the bait rather than the prey. As the bait, McJAZ6 only interacted with McJAZ2, but no interaction was detected using McJAZ6 as the prey. In addition, the rest of combinations including McJAZ7 and other McJAZ proteins failed to form heterodimers, except for a heterodimeric interaction between McJAZ7 and McJAZ2 that was observed regardless of whether McJAZ7 was used as the prey or the bait. These results suggest the existence of homo- or heterodimeric interactions among McJAZ proteins in *M. canadensis*.

## 3. Discussion

JAs play diverse functions across the JA signaling pathway in plants [8]. Notably, JAZ proteins, core negative regulators of JA signaling, are widely reported to play a vital role in JA-mediated biological processes including plant growth, defense against pathogen infection, insect attack, abiotic stress, and plant specialized metabolism [20,41,73]. To date, JAZ family proteins have been systematically identified in various plants including *A. thaliana* [33], *T. aestivum* [37], *H. brasiliensis* [39], and *L. aurea* [70]. However, research regarding the *JAZ* gene family has not yet been reported in the economically and medicinally important plant species *M. canadensis*. Based on the available transcriptome data [67], we performed a comprehensive identification of JAZ proteins in *M. canadensis* and further investigated their sequence characteristics and protein structure, evolutionary relationships, expression profiles, subcellular locations, and protein-protein interactions. This work provided a basis for subsequent functional analysis of *JAZ* genes to deepen our understanding of JA signaling in the *Lamiaceae* family, especially in *M. canadensis*.

We isolated seven *McJAZ* genes from *M. canadensis*, and found that the length of CDs in the *McJAZ* gene family ranged from 372 to 1041 bp (Figure 1 and Table 1), which was in line with the reported CDs length in the *AtJAZ* gene family [19]. Moreover, most McJAZ proteins were basic proteins with pI values greater than 7, except for McJAZ2 with a pI value of 6.12, which was attributed to a higher proportion of acidic amino acids in McJAZ2 such as 21 aspartic acids and 12 glutamic acids than those in the rest of McJAZ proteins, and this phenomenon was ubiquitous in JAZ proteins of other plants like *Camellia sinensis* [74]. In general, JAZ protein at least contains one conserved TIFY and a typical Jas domain at its N- and C-terminal, and some JAZ proteins including *Arabidopsis* AtJAZ7 and AtJAZ8 have an LxLxL type of EAR motif at the N-terminus [33]. Here, although the amino acid similarity among McJAZ proteins is low (Figure 2 and Supplemental Table S1), each identified McJAZ protein contained one conserved N-terminus TIFY and a C-terminus Jas domain, which further indicated evolutionary conservation of these structures. Furthermore, McJAZ6 and McJAZ7 have an additional EAR motif (LxLxL) in the N-terminus (Figure 2). TIFY has dual functions in regulating homo- and heterodimeric interactions among different JAZ proteins, and recruiting TPL/TPRs by interacting with NINJA protein [22]. The Jas domain is responsible for interacting with COI1 and various TFs including the core regulator MYC2 to complete the JA response [19], and is involved in subcellular localizations of JAZ proteins [75]. The EAR motif in some JAZ proteins is used to directly recruit the TPL-coupled inhibitory complex to attenuate JA response in the absence of NINJA [36]. Given that protein function is tightly associated with conserved domain structure, all of these results suggest that these seven McJAZ proteins may share some common strategy to regulate the perception of JA and JA response in *M. canadensis*, much like JAZ proteins in different plant species. Notably, the Jas domain of the JAZ protein usually contained a conserved sequence (SLX_2_FX_2_KRX_2_RX_5_PY), but our results showed that McJAZ2 has a truncated Jas motif (DLPIARKNSLARFLEKRRDR) without the X_5_PY sequence (Figure 2). Previous studies found that some deviation existed in the Jas domain owing to alternative splicing (AS). For example, AtJAZ10.3, a splice variant of AtJAZ10 protein in *Arabidopsis*, has a truncated Jas motif (DLPIARRKSLQRFLEKRKER) lacking LVSTSPY (X_5_PY) sequence in the whole Jas domain of AtJAZ10.1 [76]. Thus, whether McJAZ2 is a splice variant like AtJAZ10.3 merits further investigation.

The tissue expression patterns of target genes are closely tied to gene function. Specifically, *JAZ* family genes were constitutively and differentially expressed in different tissues of various plants such as wheat [37], rubber tree [39], and *L. aurea* [70]. Furthermore, the widespread expression of *JAZ* genes in different plant tissues is involved in regulating plant growth and development [1,41]. Here, we found that seven *McJAZ* genes were constitutively expressed in the tested tissues, and showed a variable expression profile (Figure 4). These results suggest that different *McJAZ* genes may play an important role in regulating the growth and development of different tissues in *M. canadensis*. It is intriguing that seven *McJAZ* genes showed the highest expression in flowers over all other tissues studied (Figure 4). Among these, the transcripts of *McJAZ5/6/7* reached 33-fold, 180-fold and 70-fold within flowers relative to root tissue. Moreover, earlier reports showed that *JAZ* genes played roles in flower and stamen development in *Arabidopsis* and in rice [1,19]. Thus, *McJAZ* genes may share similar functions with the above-mentioned *JAZ* genes in flower development. In addition, we found that *McJAZ1/2/4/5/6* showed similar expression patterns (Figure 4B), which, taken with the conservation of TIFY and Jas domains among them, suggests some functional redundancy in the *McJAZ1/2/4/5/6* in *M. canadensis*.

The defense-related hormones JA and ABA induce fluctuation in transcript abundance of *JAZ* genes in various plants, and this critical transcriptional regulation of *JAZ* genes is connected with the potential roles of JAZ including JA- and ABA-mediated plant development, defense, and specialized metabolism [19,29,37,38,45,47]. For instance, JA treatment and other environmental elicitors rapidly induce *JAZ* gene expression, which in turn attenuates response to JA [20]. In addition, *OsJAZ1* was induced by ABA and involved in negative regulation of drought tolerance in rice [47]. Here, the seven studied *McJAZ* genes were significantly upregulated after MeJA treatment compared with control, further pointing to their probable involvement in JA signaling. Of them, *McJAZ1*, *McJAZ3*, *McJAZ6*, and *McJAZ7* showed a short-term response to MeJA treatment at the major time points of 1 and 3 h, whereas *McJAZ2*, *McJAZ4*, and *McJAZ5* showed a continuous response to MeJA treatment from 1 to 24 h (Figure 5A), suggesting that the transcription of *McJAZ* genes may fall into distinct regulatory pathways. For ABA treatment, only *McJAZ4* expression was not affected by ABA treatment, the relative expression levels of *McJAZ1*, *McJAZ3*, and *McJAZ7* were slightly depressed, and *McJAZ2*, *McJAZ5*, and *McJAZ6* genes were up-regulated in a time-point dependent manner (Figure 5B). Notably, cross-talk between JA and ABA has been reported in plants [29]. Our study shows that *McJAZ2*, *McJAZ5*, and *McJAZ6* are also co-induced by MeJA and ABA, indicating that these *McJAZ* genes may regulate cross-talk between these two phytohormones. In addition, studies show that the *JAZ* family genes exhibited varied expression in response to one or more abiotic stressors in different plants. *JAZ* family genes are differentially induced by drought, salt, and low temperature in wheat, rice, *Camellia sinensis*, and *Brassica rapa*, and upregulation of these genes is involved in salt and drought tolerance [37,38,74,77]. Here, we found that the identified seven *McJAZ* genes showed differential upregulation to cope with drought stress, and *McJAZ2*, *McJAZ5*, *McJAZ6*, *McJAZ7* showed an intense response to drought treatment (Figure 6A). Furthermore, *McJAZ2*, *McJAZ5*, *McJAZ6*, *McJAZ7* were upregulated after salt treatment (Figure 6B), suggesting that *McJAZ* genes likely played important roles in resistance to one or more abiotic stressors. In addition, abiotic stress-responsive *McJAZ* genes were simultaneously upregulated by JA and ABA treatments, suggesting that *McJAZ* genes may respond to different stresses via diverse phytohormone signaling pathways in *M. canadensis*.

**Figure 3:**
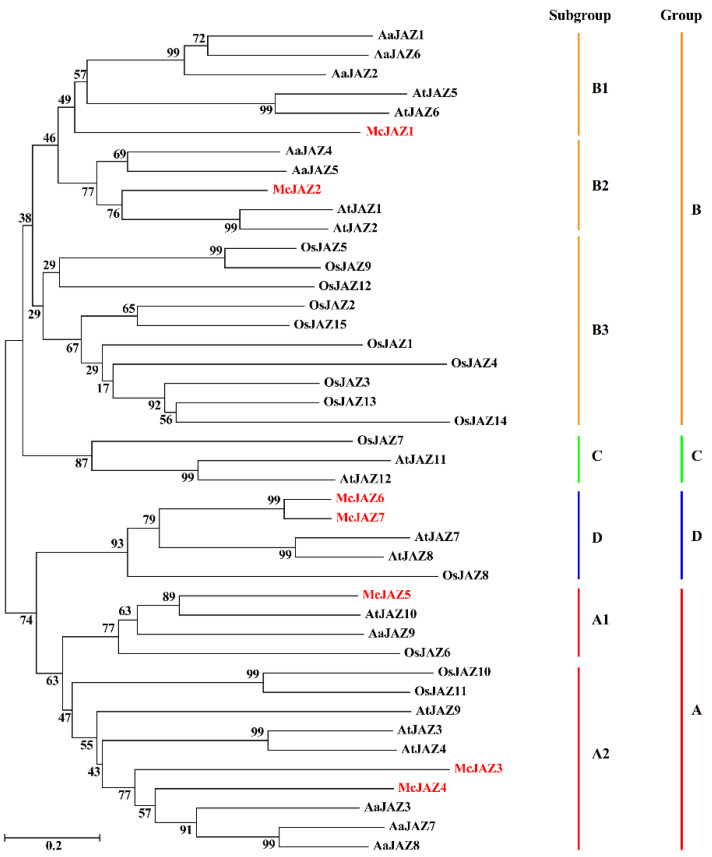
**Phylogenetic analysis of JAZ proteins from *M. canadensis* and other species.** JAZ amino acid sequences from *M. canadensis* were aligned with those from *A. thaliana* (eudicot), *O. sativa* (monocot) and *A. annua* (eudicot) using Clustal X2, and a neighbor-joining (NJ) phylogenetic tree was constructed using MEGA5.1. Bootstrap values indicate the percentage among 2000 replicates. JAZ proteins of *M. canadensis* were labeled with red font. *Mc*, *M. canadensis*. *At*, *A. thaliana*, *Os*, *O. sativa*, *Aa*, *A. annua*. The accession numbers or locus name of each JAZ amino acid sequence shown is as follows: *A. thaliana* AtJAZ1, NP973862.1, AtJAZ2, AAP13409.1, AtJAZ3, NP001078174, AtJAZ4, AAX55088.1, AtJAZ5, AAO00903.1, AtJAZ6, AAL15195.1, AtJAZ7, AAR24741.1, AtJAZ8, ABG48454.1, AtJAZ9, AAM10238.1, AtJAZ10, NP001154713.1, AtJAZ11, AAU15160.1, AtJAZ12, AAK93690.1; *O. sativa* OsJAZ1, Os04g55920, OsJAZ2, Os07g05830, OsJAZ3, Os08g33160, OsJAZ4, Os09g23660, OsJAZ5, Os04g32480, OsJAZ6, Os03g28940, OsJAZ7, Os07g42370, OsJAZ8, Os09g26780, OsJAZ9, Os03g08310, OsJAZ10, Os03g08330, OsJAZ11, Os03g08320, OsJAZ12, Os10g25290, OsJAZ13, Os10g25230, OsJAZ14, Os10g25250, OsJAZ15, Os03g27900; *A. annua*, AaJAZ1, AJK93412.1, AaJAZ2, AJK93413.1, AaJAZ3, AJK93414.1, AaJAZ4, AJK93415.1, AaJAZ5, APR73266.1, AaJAZ6, APR73267.1, AaJAZ7, APR73268.1, AaJAZ8, APR73269.1, AaJAZ9, APR73270.1.

In plants, JAZ proteins participate in JA signaling pathways by interacting with JA receptor COI1 or themselves [19,34]. In our study, some of the McJAZ proteins were found to interact with McCOI1a in a COR-dependent manner (Figure 8). Furthermore, McJAZ proteins showed variable capacities to form homo- or heterodimers via interacting with each other in a COR-independent manner (Figure 9). These results suggest that the functions of *McJAZ* gene in *M. canadensis* might be similar to those in *Arabidopsis*. Previous studies have shown that AtJAZ1 can interact with AtCOI1 or SlCOI1 in the presence of COR [32]. However, the identified McJAZ proteins only interacted with McCOI1a rather than AtCOI1 in the presence of COR, suggesting that regulation of the interactions of JAZ-COI1 may differ across species. In addition, the phylogenetic relationship among the *JAZ* genes reveals the putative function of them as homologous genes often share similar biological roles. In this study, we found that McJAZ1 and McJAZ2 clustered with AtJAZ1/2, or AtJAZ5/6 into the subgroup B2 or B1. McJAZ3, McJAZ4, and McJAZ5 clustered with AtJAZ3/4 or AtJAZ10 into the subgroup A2 or A1. McJAZ6/7 clustered with AtJAZ7/8 into the group D (Figure 3). It was clear that AtJAZ proteins typically interacted with AtMYC2 to regulate diverse physiological functions including plant metabolism and defense [78,79]. Additionally, AtJAZ3 interacted with AtSPL9 to regulate plant resistance to insect herbivores [80]. AtJAZ1/4/9 interacted with AtICE1/2 to regulate freezing tolerance in *Arabidopsis* [25]. AtJAZ1/8/11 interacted with and attenuated the transcriptional functions of MYB21 and MYB24 to regulate stamen development [81]. AtJAZ1/2/5/6/8/9/10/11 also regulated JA-mediated anthocyanin biosynthesis and trichome development by interacting with the WD-Repeat/bHLH/MYB complexes in *Arabidopsis* [14]. Given the functional specificity, diversity, and redundancy of *AtJAZ* genes in *Arabidopsis* [82], McJAZ proteins may regulate plant development, defense, and specialized metabolism including EO biosynthesis by interacting with the as-yet unidentified McMYC2 or additional TFs in *M. canadensis* (Figure 10). Further investigation needs to be conducted to determine the complete regulatory network of McJAZ proteins.

Notably, JA treatment promoted biosynthesis of EO in two *Mentha* plants [58,67], and EO from *Mentha* plants could be used to control plant bacterial disease caused by *A. tumefaciens* in tomato [83], enhance neighboring plants’ resistance to insect herbivores [56], and act as a natural insecticide for control of insects including *Tribolium castaneum*, *Lasioderma serricorne*, and *Liposcelis bostrychophila* adults [84]. This suggests that manipulation of JA signaling in *Mentha* plants might have dual roles in regulating EO biosynthesis and affecting the anti-insect or anti-pathogen ability of neighboring plants. JAZ proteins, the key components of the JA signaling pathway, acted as important regulators involved in mediating biosynthesis of specialized metabolism such as anthocyanin [14,85], tanshinone [49], and artemisinin [24] through employing the different regulatory mechanisms. However, the detailed molecular mechanisms of JA-mediated EO biosynthesis by *McJAZ* genes or a McJAZ-related network remain uninvestigated within the mint family. Thus, subsequent research into functional analysis of *McJAZ* genes or McJAZ-associated regulatory networks in *Mentha* plants will deepen our understanding of the regulatory mechanisms of JA-mediated EO biosynthesis, and help us to develop efficient strategies for genetic improvement of EO content, and even enhance plant defense against pathogen infection, herbivorous insect attack, and abiotic stress in *M. canadensis*.

## 4. Materials and Methods

### 4.1. Plant Materials and Treatments

*M. canadensis* was conserved and planted at the Germplasm Nursery in the Institute of Botany, Jiangsu Province, and Chinese Academy of Sciences, Nanjing, Jiangsu Province [67], for use in each assay described here. Cuttings of *M. canadensis* were propagated by inserting into water until roots emerged, and then planted into plastic pots containing a mixture of nutrient soil and vermiculite (2:1, *v*/*v*). For in vitro culture of *N. benthamiana*, seeds were sown on soil and 10-day-old seedlings were transplanted into plastic pots containing a mixture of nutrient soil and vermiculite (2:1, *v*/*v*). Next, *M. canadensis* and *N. benthamiana* plants were grown in a light chamber at 23 ± 2 °C with the desired light regime, and a 16-h light/8-h dark photoperiod.

For hormone and salt stress treatments, two-week-old *M. canadensis* seedlings were sprayed with 100 μM MeJA (Sigma-Aldrich (Shanghai) Trading Co. Ltd., Shanghai, China), 20 μM ABA (Sigma-Aldrich (Shanghai) Trading Co. Ltd., Shanghai, China), and 150 mM NaCl for 1, 3, 6, 12, and 24 hours (h) according to methods adapted from previous studies [12,86]. For drought treatment, two-week-old hydroponically cultured *M. canadensis* seedlings were exposed to dry filter paper for 1, 3, 6, 12, and 24 h at room temperature according to methods adapted from previous studies [87]. Untreated plants were used as the control (labeled as CK 0 h). Then, leaves of the control and treated seedlings, of matching positions, were collected at the indicated time point and frozen in liquid nitrogen and stored at −80 °C for further analysis.

### 4.2. Isolation and Cloning of M. canadensis McJAZ Family Genes

The isolation of *M. canadensis McJAZ* genes were performed as described previously [88]. In short, the transcriptome data (SRP132644) of *M. canadensis* was downloaded from the National Center for Biotechnology Information (NCBI, https://www.ncbi.nlm.nih.gov/, accessed on 20 March 2020). The well-known 12 *A. thaliana* [34], and 9 *A. annua* JAZ protein sequences were downloaded from TAIR (https://www.arabidopsis.org/, accessed on 20 January 2021) or the annotated *A. annua* genome [89], respectively. Then, each of them was employed as queries to search for JAZ proteins in *M. canadensis* protein databases by the local BLASTP method with an e-value of 1 × 10^−5^. The screened sequences were submitted to ORF Finder (https://www.ncbi.nlm.nih.gov/orffinder, accessed on 15 March 2021) to gain the complete coding sequences, and the candidate sequences were subjected to SMART (http://smart.embl-heidelberg.de/, accessed on 25 April 2021) tools with default parameters to substantiate the existence of the conserved TIFY and Jas domains [33], and determine their precise positions. The candidate *McJAZ* gene sequences with both TIFY and Jas domains were selected for further analysis.

To clone the *McJAZ* genes, the young leaves of two-week-old *M. canadensis* were collected for RNA extraction using the reagent Trizol (Cat^#^ 9108, Takara Biomedical Technology (Beijing) Co., Ltd., Beijing, China), and cDNA was synthesized using PrimeScript RT reagent Kit with gDNA Eraser (RR047A, Takara Biomedical Technology (Beijing) Co., Ltd., Beijing, China). The candidate *McJAZ* genes were amplified using PCR with defined primers (Supplemental Table S2) in a 25 μL volume including 1 μL 5x cDNA template, 0.5 μL of each forward and reverse primer, 5 μL 5x PrimeSTAR DNA Polymerase buffer, 2 μL PrimeSTAR^®^ DNA Polymerase, and 16 μL sterile ddH_2_O. The amplification procedure was as follows: 95 °C for 10 min followed by 35 cycles of denaturation for 15 s at 95 °C, annealing at designated temperature for 20 s, and extension at 72 °C for 1 min per kilobase (kb). Subsequently, the full-length coding sequences of *McJAZ* genes were gel-purified using Gel DNA Mini Purification Kit (TSINGKE Biotechnology, Beijing, China), and the purified amplicons were ligated with pClone007 Blunt Simple vector (TSV-007B, TSINGKE Biotechnology, Beijing, China), and then the resulting products were transformed into *Escherichia coli* DH5α and sequenced in TSINGKE Biotechnology Co., Ltd (TSINGKE Biotechnology, Chengdu, China).

### 4.3. Sequence Analysis and Phylogenetic Tree Analysis

The sequence and physio-chemical properties of candidate McJAZ proteins were predicted using the online ExPASy server (http://us.expasy.org/tools, accessed on 3 May 2021) [90], including the length of amino acid (aa) sequences, molecular weight (*M*_W_), and theoretical protein isoelectric points (pIs). The CELLO v.2.5 tool (http://cello.life.nctu.edu.tw/, accessed on 30 April 2021) was employed to predict the subcellular localization of candidate McJAZ proteins.

Multiple sequence alignment analysis of the seven McJAZ proteins was performed to analyze the characteristics of the conserved sequences using Clustal X2 software with default settings [91]. Meanwhile, multiple sequence alignment of the pairwise McJAZ proteins to identify the amino acid sequence similarity was done using DNAMAN 6.0 (Lynnon Biosoft, Quebec City, QC, Canada) software with default parameters. The frequency of the conserved amino acids at each site within the TIFY and Jas domain was analyzed using online MEME tools (http://meme-suite.org/tools/meme, accessed on 8 May 2021) using the default settings. The composition and position of the conserved motifs, and the sequence logos of the TIFY and Jas domains were created by online MEME tools. Next, to check out the evolutionary relationships of McJAZ family proteins, the JAZ protein sequences including 12 *A. thaliana* [20], 15 *O. sativa* [38] and 9 *A. annua* [24] homologous proteins were selected and acquired from the databases Phytozome v12.1 (https://phytozome.jgi.doe.gov/, accessed on 20 January 2021) and NCBI, respectively. The MEGA 5.0 program was adopted to construct the phylogenetic tree using a neighbor-joining (NJ) approach with the following parameters: Pairwise deletion and 2000 bootstrap replicates [92].

### 4.4. Subcellular Localization of McJAZ Proteins

The full-length coding sequences of each *McJAZ* gene without its stop codon was PCR-amplified using gene-specific primers (Supplemental Table S2), and the amplicons were subcloned into the N-terminal side of the pCAMBIA1300 vector containing the green fluorescent protein (GFP) reporter gene to produce the recombinant fusion construct pCAMBIA1300-McJAZs-GFP under the control of the CaMV 35S promoter. Then the fusion constructs were transformed into *Agrobacterium* strains GV3101 through the conventional freezing-thawing method, and the GV3101 strains harboring each pCAMBIA1300-McJAZs-GFP or empty pCAMBIA1300-GFP were transiently infiltrated into 5-week-old *N. benthamiana* leaves according to previous report [24]. GFP signals were detected 2 to 3 days after infiltration using a confocal laser-scanning microscope (Leica TCS SP5-II, Wetzlar, Germany).

### 4.5. RNA Extraction and Quantitative Real-Time PCR (qRT-PCR) Analysis

**Figure 4:**
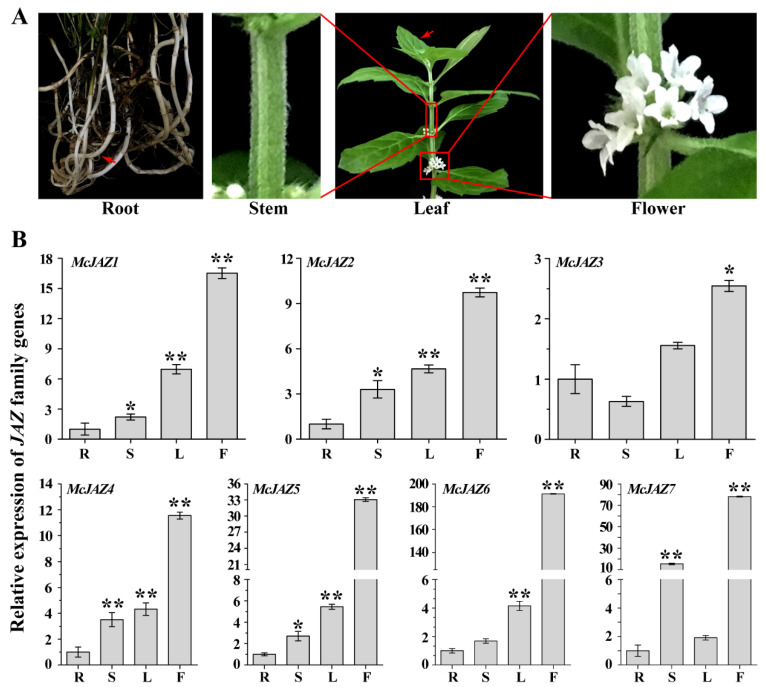
**Tissue-specific expression patterns of *M. canadensis McJAZ* genes.** (**A**) The different tissues used for total RNA extraction, including Root (R), Stem (S), Young Leaf (L) and Flower (F). The red arrow indicates the position at which root and leaf tissues were collected. (**B**) Quantitative real-time (qRT)-PCR analysis of each *McJAZ* gene expression in the indicated *M. canadensis* tissues. The expression level of each *McJAZ* gene was normalized to *β-actin* gene and relative expression values are compared in root. Data are presented as means ± SE of three independent replicates, and significant differences between the expression level of each *McJAZ* gene in root tissue versus other tissue was analyzed by Student’s *t*-test (* *p* < 0.05 or ** *p* < 0.01).

**Figure 5:**
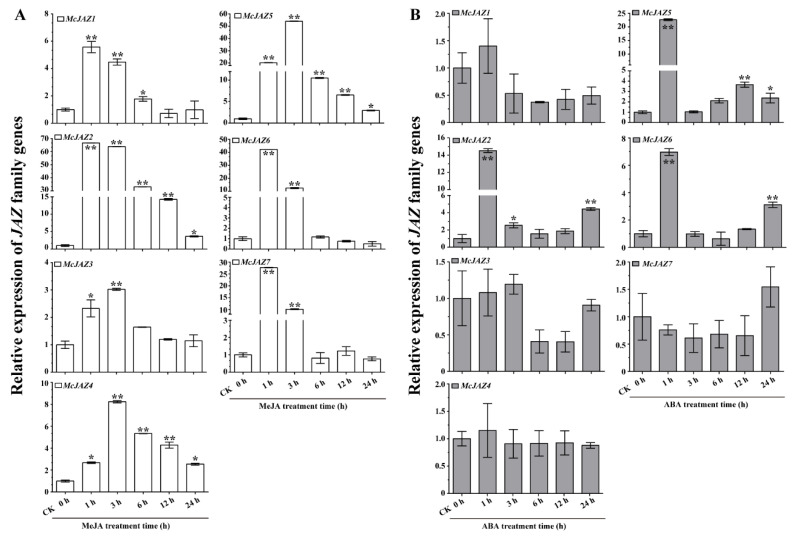
**Expression profiles of *McJAZ* genes under phytohormone MeJA and ABA treatments**. Quantitative real-time (qRT)-PCR analysis of the expression levels of *McJAZ1*, *McJAZ2*, *McJAZ3*, *McJAZ4*, *McJAZ5*, *McJAZ6*, and *McJAZ7* in the two-week-old *M. canadensis* seedlings under 100 μM Methyl Jasmonate (MeJA) (**A**) or 20 μM Abscisic Acid (ABA) (**B**) treatments for 0, 1, 3, 6, 12, and 24 h, respectively. The relative expression level of each *McJAZ* gene was normalized to *β-actin* gene and compared with CK (0 h) as reference using the 2^−∆∆Ct^ method. Data are presented as means ± SE of three independent replicates from three propagated cuttings. Asterisks indicate significant differences between the expression level of each *McJAZ* gene in CK (0 h) versus other time points were analyzed by Student’s *t*-test (* *p* < 0.05 or ** *p* < 0.01). CK (0 h) represents an independent control under identical conditions.

**Figure 6:**
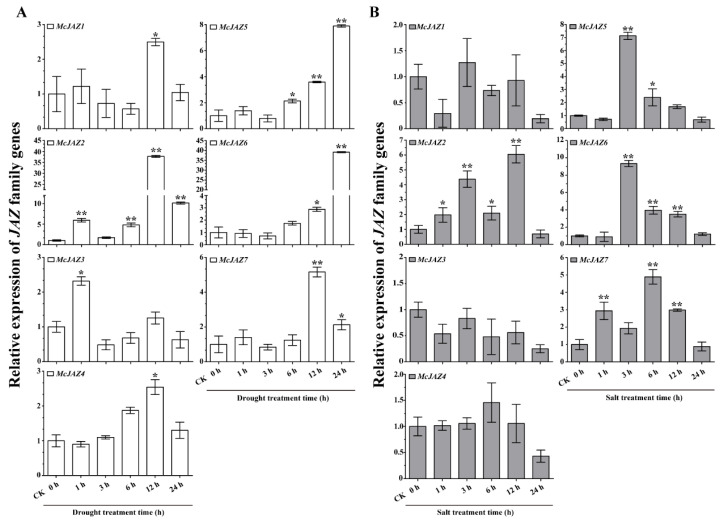
**Expression profiles of *McJAZ* genes under drought and salt stress treatments.** Quantitative real-time (qRT)-PCR analysis of the expression levels of *McJAZ1*, *McJAZ2*, *McJAZ3*, *McJAZ4*, *McJAZ5*, *McJAZ6*, and *McJAZ7* in the two-week-old *M. canadensis* seedlings under drought (**A**) or 150 mM NaCl (**B**) treatments for 0, 1, 3, 6, 12, and 24 h. The relative expression level of each *McJAZ* gene was normalized to *β-actin* gene and compared with CK (0 h) as reference using the 2^−∆∆CT^ method. Data are presented as means ± SE of three independent replicates from three propagated cuttings. Asterisks indicate significant differences between the expression level of each *McJAZ* gene in CK (0 h) versus other time points were analyzed by Student’s *t*-test (* *p* < 0.05 or ** *p* < 0.01). CK (0 h) represents an independent control under identical conditions.

**Figure 7:**
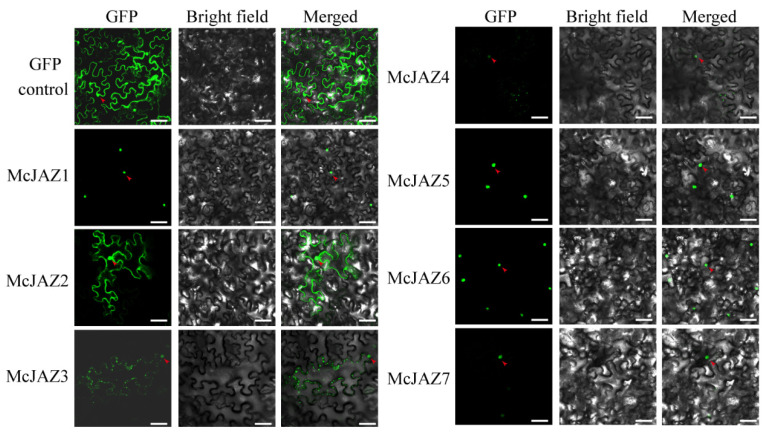
**Subcellular localization analysis of McJAZ proteins in *N. benthamiana* leaf cells.** The GFP-tagged McJAZ fusion proteins and empty green fluorescence protein (GFP) alone were transiently expressed in the epidermal cells of *N. benthamiana* leaf under the control of the CaMV 35S promoter. GFP fluorescence was detected at two days after infiltration in *N. benthamiana* leaves by confocal laser microscopy. GFP alone is labeled as GFP control. The red arrow indicates the nucleus within each image. Bar = 20 μm.

**Figure 8:**
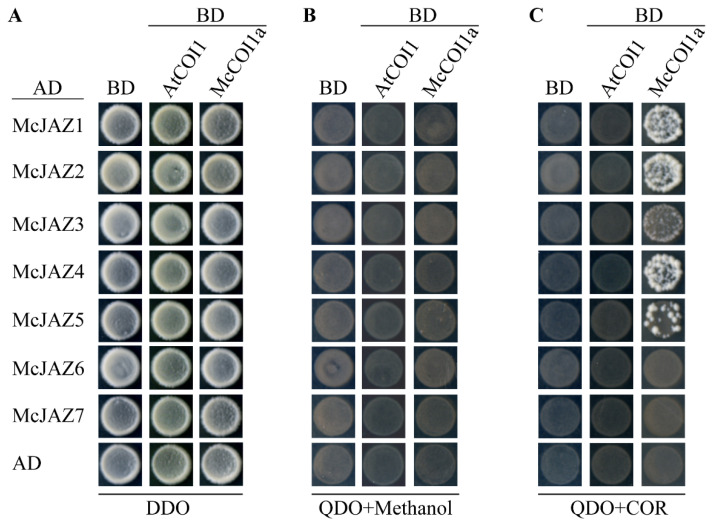
**Yeast two**-**hybrid interactions between McJAZ proteins and McCOI1a or AtCOI1.** The co-transformed yeast cells harboring the indicated plasmid combinations were inoculated in liquid medium SD/-Leu/-Trp (DDO), and then placed onto control medium SD/-Leu/-Trp (DDO) (**A**), selection medium SD/-Leu/-Trp/-His/-Ade (QDO) plus Methanol (**B**), and selection medium SD/-Leu/-Trp/-His/-Ade (QDO) containing 35 μM coronatine (COR) (**C**). The empty pGADT7 (AD) and pGBKT7 (BD) were used as negative controls. These data represent three independent experiments, and representative photos were taken after 4 days of incubation at 30 °C.

**Figure 9:**
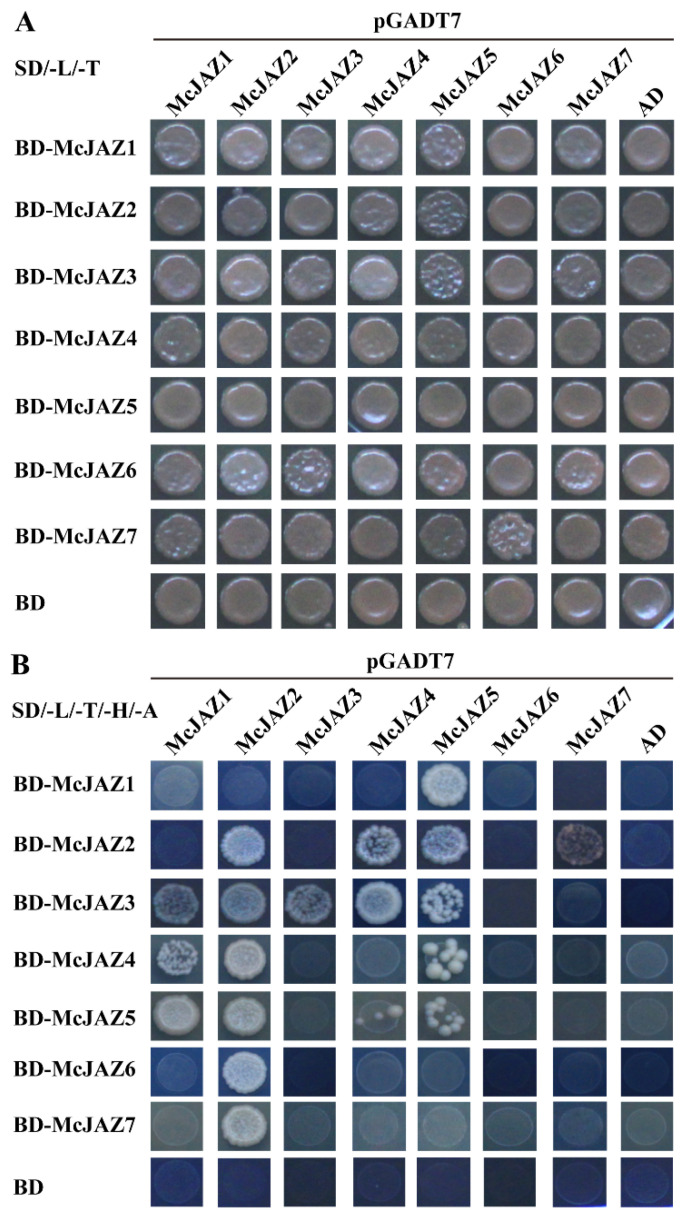
**Yeast two**-**hybrid interactions among different McJAZ proteins.** Yeast cells transformed with different combinations of constructs containing McJAZ proteins fused with the DNA binding domain (BD-McJAZ1, BD-McJAZ2, BD-McJAZ3, BD-McJAZ4, BD-McJAZ5, BD-McJAZ6, and BD-McJAZ7), McJAZ proteins fused with the activation domain (AD-McJAZ1, AD-McJAZ2, AD-McJAZ3, AD-McJAZ4, AD-McJAZ5, AD-McJAZ6, and AD-McJAZ7), and the pGBKT7 (BD) alone, and the pGADT7 (AD) alone were inoculated in liquid medium and then placed on control medium SD/-Leu/-Trp (SD/-L/-T) (**A**), and selective media SD/-Leu/-Trp/-His/-Ade (SD/-L/-T/-H/-A) (**B**). The empty vectors (AD and BD) were transformed into yeast and used as negative controls. These data represent three independent experiments, and representative pictures were taken after 4 days of incubation at 30 °C.

**Figure 10:**
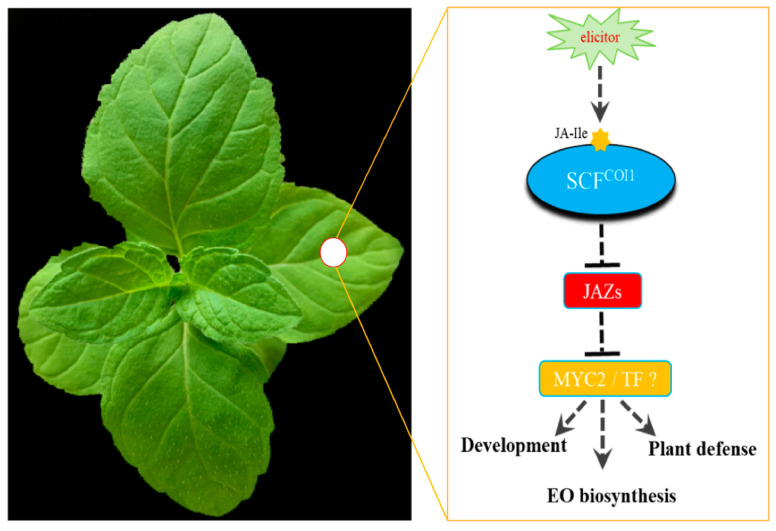
**A proposed working model of how JA signaling module regulates diverse biological processes in response to JA signaling in *M. canadensis***. A simple model showing the putative role of the JA signaling module in controlling diverse biological processes in *M. canadensis*. McCOI1, a homolog of the Arabidopsis F-box protein CORONATINE INSENSITIVE 1 (AtCOI1) (Xie et al., 1998), acts as a putative receptor for the active molecule JA-isoleucine (JA-Ile). Introduction of the exogenous elicitor induces the production of JA-Ile, which is perceived by McCOI1 to form the activated McCOI1-JA-Ile complex that subsequently interacts with the repressor, JASMONATE ZIM DOMAIN (JAZ) proteins, and represses its transcriptional inhibitory ability by degradation of McJAZ proteins through the SCFCOI1-dependent 26S proteasome pathway (Chini et al., 2007; Howe et al., 2018). Next, degradation of the McJAZ proteins relieves repression of downstream transcription factors (TFs) including unidentified McMYC2 or other McTFs, which are involved in regulating plant development, plant defense, and essential oil (EO) biosynthesis in *M. canadensis*. A photograph of normal growing *M. canadensis* was used as the model; shown at left.

To study tissue expression patterns, different tissues of 3-month-old *M. canadensis*, including roots, stems, young leaves, and flowers were harvested and stored at −80 °C. Total RNA of the different tissues (labeled in Figure 4A) and the aforementioned samples was isolated using the total RNAprep pure Extraction Kit (Tiangen Biotech, Beijing, China) according to the manufacturer’s recommendations. cDNA was synthesized from 1.0 μg total RNA using the PrimeScript 1st Strand cDNA Synthesis Kit (Takara Biomedical Technology (Beijing) Co., Ltd., Beijing, China) according to the manufacturer’s instructions. The qRT-PCR was performed using the SYBR Premix Ex Taq II Kit (Tiangen Biotech, Beijing, China) according to the manufacturer’s instructions and methods previously described [93]. All PCR reactions were performed on at least three replicates under identical conditions. Expression level of each gene was normalized to the expression of *β-actin* gene (KM044035) used as internal control in the mint family in the previous study [61], and the relative mRNA expression for each gene was calculated using the 2^−∆∆Ct^ method [94]. All primers are listed in Supplemental Table S2.

### 4.6. Yeast Two-Hybrid (Y2H) Experiments

Yeast two-hybrid assays were used to test the paired physical interactions among McJAZ proteins, or the interactions between McJAZ proteins and AtCOI1, or its homolog McCOI1a. The full-length coding sequences of *McJAZ* genes were cloned into the prey vector pGADT7 (Activation domain, AD) or bait vector pGBKT7 (Binding domain, BD) (Takara, Japan), respectively, and the full-length coding sequences of *McCOI1a* and *AtCOI1* were amplified using gene-specific primers (Supplemental Table S2), and inserted into the bait vector pGBKT7 (BD). The indicated combinations were co-transformed into the yeast strain AH109 using the lithium acetate method according to the manufacturer’s instructions (Cat^#^ 630440, Takara Biomedical Technology (Beijing) Co., Ltd., Beijing, China), and bait-only or prey-only was tested with empty AD or BD as negative controls.

To investigate protein-protein interactions, the transformed AH109 yeast cells were first cultivated in liquid control medium SD/-Leu/-Trp (DDO) over 24 h, and then 5 μL of each yeast suspension was taken to inoculate the solid control medium SD/-Leu/-Trp (DDO) and selection medium SD/-Leu/-Trp/-Ade/-His (QDO) with or without 35 μM coronatine (COR), a structural and functional mimic of JA-Ile obtained from several strains of *Pseudomonas syringae* according to previous studies [19,72,95]. The yeast cells were grown at 30 °C for 4 days before imaging.

### 4.7. Statistical Analysis

All experiments were conducted with at least three biological replicates. Significant differences between the control versus each treatment were analyzed by Student’s *t*-test using GraphPad Prism 5 software. The values are mean ± standard error (SE) for replicates in each group. * *p* values ≤ 0.05 or ** *p* values ≤ 0.01 were considered as significant or extremely significant.

### 4.8. Accession Numbers

The gene sequences data mentioned in this article can be found in the NCBI database under the accession numbers: McJAZ1 (MZ229995), McJAZ2 (MZ229996), McJAZ3 (MZ229997), McJAZ4 (MZ229998), McJAZ5 (MZ229999), McJAZ6 (MZ230000), McJAZ7 (MZ230001), and AtCOI1 (AT2G39940).

## Figures and Tables

**Table 1:** Molecular characteristics of *McJAZ* genes in *M. canadensis*.

Gene Name	Genbank Accession No. ^1^	Length CDs ^2^	Amino Acids ^3^	*M*_W_ (kDa) ^4^	pI ^5^	TIFY Motif ^6^	Jas Motif (aa) ^7^	Protein Localization
*McJAZ1*	MZ229995	726	241	26.30	8.98	TIFYSG	196–221	Nuclei
*McJAZ2*	MZ229996	522	173	18.35	6.12	TIFYGG	156–173	Nuclei
*McJAZ3*	MZ229997	1041	346	36.89	9.75	TIFYNG	294–319	Nuclei
*McJAZ4*	MZ229998	789	262	28.09	9.17	TIFYGG	230–249	Nuclei
*McJAZ5*	MZ229999	549	182	19.93	9.76	TIFYNG	158–182	Nuclei
*McJAZ6*	MZ230000	378	125	14.02	9.96	TIFYNG	93–121	Nuclei
*McJAZ7*	MZ230001	372	123	13.87	9.77	TIFYNG	91–119	Nuclei

^1^ Genbank Accession number; ^2^ the length of coding sequences; ^3^ the length of amino acid sequences; ^4^ the molecular weight of protein (kDa); ^5^ the isoelectric point (pI); ^6^ the core sequence feature of TIFY motif; ^7^ the position of Jas domain within the amino acid sequences; aa: amino acid.

## Data Availability

All data in the present research are available in the public database as referred in the Material and Method section.

## Supplementary Materials

The following are available online at https://www.mdpi.com/article/10.3390/ijms22168859/s1.

## Abbreviations

MeJA: Methyl Jasmonate; ABA: Abscisic Acid; qRT-PCR: Quantitative Real-Time Polymerase Chain Reaction; Y2H: Yeast Two-Hybrid; GFP: Green Fluorescent Protein.

## References

[1] Yuan Z., Zhang D. (2015). Roles of jasmonate signaling in plant inflorescence and flower development. Curr. Opin. Plant Biol..

[2] Browse J. (2005). Jasmonate: An oxylipin signal with many roles in plants. Vitam. Horm..

[3] Huang H., Liu B., Liu L., Song S. (2017). Jasmonate action in plant growth and development. J. Exp. Bot..

[4] Han X., Zhang M., Yang M., Hu Y. (2020). *Arabidopsis* JAZ proteins interact with and suppress RHD6 transcription factor to regulate jasmonate-stimulated root hair development. Plant Cell.

[5] Wasternack C., Hause B. (2013). Jasmonates: Biosynthesis, perception, signal transduction and action in plant stress response, growth and development. An update to the 2007 review in Annals of Botany. Ann. Bot..

[6] Zhang Y., Turner J.G. (2008). Wound-induced endogenous jasmonates stunt plant growth by inhibiting mitosis. PLoS ONE.

[7] Reinbothe C., Springer A., Samol I., Reinbothe S. (2009). Plant oxylipins: Role of jasmonic acid during programmed cell death, defence and leaf senescence. FEBS J..

[8] Howe G.A., Major I.T., Koo A.J. (2018). Modularity in jasmonate signaling for multistress resilience. Ann. Rev. Plant Biol..

[9] Wang J., Song L., Gong X., Xu J., Li M. (2020). Functions of jasmonic acid in plant regulation and response to abiotic stress. Int. J. Mol. Sci..

[10] Basso V., Veneault-Fourrey C. (2020). Role of jasmonates in beneficial microbe-root interactions. Methods Mol. Biol..

[11] Vijayan P., Shockey J., Lévesque C.A., Cook R.J., Browse J. (1998). A role for jasmonate in pathogen defense of *Arabidopsis*. Proc. Natl. Acad. Sci. USA.

[12] Ma Y.N., Xu D.B., Yan X., Wu Z.K., Kayani S.I., Shen Q., Fu X.Q., Xie L.H., Hao X.L., Hassani D. (2021). Jasmonate- and abscisic acid-activated AaGSW1-AaTCP15/AaORA transcriptional cascade promotes artemisinin biosynthesis in *Artemisia annua*. Plant Biotechnol. J..

[13] Afrin S., Huang J.-J., Luo Z.-Y. (2015). JA-mediated transcriptional regulation of secondary metabolism in medicinal plants. Sci. Bull..

[14] Qi T., Song S., Ren Q., Wu D., Huang H., Chen Y., Fan M., Peng W., Ren C., Xie D. (2011). The Jasmonate-ZIM-domain proteins interact with the WD-Repeat/bHLH/MYB complexes to regulate jasmonate-mediated anthocyanin accumulation and trichome initiation in *Arabidopsis thaliana*. Plant Cell.

[15] van der Fits L., Memelink J. (2000). ORCA3, a jasmonate-responsive transcriptional regulator of plant primary and secondary metab- olism. Science.

[16] Pauwels L., Morreel K., De Witte E., Lammertyn F., Van Montagu M., Boerjan W., Inzé D., Goossens A. (2008). Mapping methyl jasmonate-mediated transcriptional reprogramming of metabolism and cell cycle progression in cultured *Arabidopsis* cells. Proc. Natl. Acad. Sci. USA.

[17] Wasternack C., Strnad M. (2019). Jasmonates are signals in the biosynthesis of secondary metabolites -pathways, transcription factors and applied aspects—A brief review. N. Biotechnol..

[18] Ruan J., Zhou Y., Zhou M., Yan J., Khurshid M., Weng W., Cheng J., Zhang K. (2019). Jasmonic acid signaling pathway in plants. Int. J. Mol. Sci..

[19] Thines B., Katsir L., Melotto M., Niu Y., Mandaokar A., Liu G., Nomura K., He S.Y., Howe G.A., Browse J. (2007). JAZ repressor proteins are targets of the SCF^COI1^ complex during jasmonate signalling. Nature.

[20] Chini A., Fonseca S., Fernández G., Adie B., Chico J.M., Lorenzo O., García-Casado G., López-Vidriero I., Lozano F.M., Ponce M.R. (2007). The JAZ family of repressors is the missing link in jasmonate signalling. Nature.

[21] Gfeller A., Liechti R., Farmer E.E. (2010). *Arabidopsis* jasmonate signaling pathway. Sci. Signal..

[22] Pauwels L., Barbero G.F., Geerinck J., Tilleman S., Grunewald W., Pérez A.C., Chico J.M., Bossche R.V., Sewell J., Gil E. (2010). NINJA connects the co-repressor TOPLESS to jasmonate signalling. Nature.

[23] Xie D.X., Feys B.F., James S., Nieto-Rostro M., Turner J.G. (1998). COI1: An *Arabidopsis* gene required for jasmonate-regulated defense and fertility. Science.

[24] Ma Y.N., Xu D.B., Li L., Zhang F., Fu X.Q., Shen Q., Lyu X.Y., Wu Z.K., Pan Q.F., Shi P. (2018). Jasmonate promotes artemisinin biosynthesis by activating the TCP14-ORA complex in *Artemisia annua*. Sci. Adv..

[25] Hu Y., Jiang L., Wang F., Yu D. (2013). Jasmonate regulates the Inducer of CBF expression-C-repeat Binding FAC TOR/DRE Binding Factor1 cascade and freezing tolerance in *Arabidopsis*. Plant Cell.

[26] Chini A., Boter M., Solano R. (2009). Plant oxylipins: COI1/JAZs/MYC2 as the core jasmonic acid-signalling module. FEBS J..

[27] Van der Does D., Leon-Reyes A., Koornneef A., Van Verk M.C., Rodenburg N., Pauwels L., Goossens A., Körbes A.P., Memelink J., Ritsema T. (2013). Salicylic acid suppresses jasmonic acid signaling downstream of SCF^COI1^-JAZ by targeting GCC promoter motifs via transcription factor ORA59. Plant Cell.

[28] Song S., Qi T., Wasternack C., Xie D. (2014). Jasmonate signaling and crosstalk with gibberellin and ethylene. Curr. Opin. Plant Biol..

[29] Kazan K., Manners J.M. (2012). JAZ repressors and the orchestration of phytohormone crosstalk. Trends Plant Sci..

[30] Pauwels L., Goossens A. (2011). The JAZ proteins: A crucial interface in the jasmonate signaling cascade. Plant Cell.

[31] Bai Y., Meng Y., Huang D., Qi Y., Chen M. (2011). Origin and evolutionary analysis of the plant-specific TIFY transcription factor family. Genomics.

[32] Melotto M., Mecey C., Niu Y., Chung H.S., Katsir L., Yao J., Zeng W., Thines B., Staswick P., Browse J. (2008). A critical role of two positively charged amino acids in the Jas motif of *Arabidopsis* JAZ proteins in mediating coronatine- and jasmonoyl isoleucine-dependent interactions with the COI1 F-box protein. Plant J..

[33] Cuéllar Pérez A., Nagels Durand A., Vanden Bossche R., De Clercq R., Persiau G., Van Wees S.C.M., Pieterse C.M.J., Gevaert K., De Jaeger G., Goossens A. (2014). The non-JAZ TIFY protein TIFY8 from *Arabidopsis thaliana* is a transcriptional repressor. PLoS ONE.

[34] Chini A., Fonseca S., Chico J.M., Fernández-Calvo P., Solano R. (2009). The ZIM domain mediates homo- and heteromeric interac- tions between *Arabidopsis* JAZ proteins. Plant J..

[35] Thireault C., Shyu C., Yoshida Y., Aubin B., Campos M.L., Howe G.A. (2015). Repression of jasmonate signaling by a non-TIFY JAZ protein in *Arabidopsis*. Plant J..

[36] Shyu C., Figueroa P., DePew C.L., Cooke T.F., Sheard L.B., Moreno J.E., Katsir L., Zheng N., Browse J., Howe G.A. (2012). JAZ8 lacks a canonical degron and has an EAR motif that mediates transcriptional repression of jasmonate responses in *Arabidopsis*. Plant Cell.

[37] Wang Y., Qiao L., Bai J., Wang P., Duan W., Yuan S., Yuan G., Zhang F., Zhang L., Zhao C. (2017). Genome-wide characterization of JASMONATE-ZIM DOMAIN transcription repressors in wheat (*Triticum aestivum* L.). BMC Genom..

[38] Ye H., Du H., Tang N., Li X., Xiong L. (2009). Identification and expression profiling analysis of TIFY family genes involved in stress and phytohormone responses in rice. Plant Mol. Biol..

[39] Chao J., Zhao Y., Jin J., Wu S., Deng X., Chen Y., Tian W.M. (2019). Genome-wide identification and characterization of the *JAZ* gene family in Rubber Tree (*Hevea brasiliensis*). Front. Genet..

[40] Chini A., Ben-Romdhane W., Hassairi A., Aboul-Soud M.A.M. (2017). Identification of *TIFY/JAZ* family genes in *Solanum lycoper- sicum* and their regulation in response to abiotic stresses. PLoS ONE.

[41] Oblessuc P.R., Obulareddy N., DeMott L., Matiolli C.C., Thompson B.K., Melotto M. (2020). JAZ4 is involved in plant defense, growth, and development in *Arabidopsis*. Plant J..

[42] Guo Q., Yoshida Y., Major I.T., Wang K., Sugimoto K., Kapali G., Havko N.E., Benning C., Howe G.A. (2018). JAZ repressors of metabolic defense promote growth and reproductive fitness in *Arabidopsis*. Proc. Natl. Acad. Sci. USA.

[43] Monte I., Franco-Zorrilla J.M., García-Casado G., Zamarreño A.M., García-Mina J.M., Nishihama R., Kohchi T., Solano R. (2019). A single JAZ repressor controls the jasmonate pathway in *Marchantia polymorpha*. Mol. Plant.

[44] Hanif M., Rahman M.U., Gao M., Yang J., Ahmad B., Yan X., Wang X. (2018). Heterologous expression of the *Grapevine JAZ7* gene in *Arabidopsis* confers enhanced resistance to powdery mildew but not to *Botrytis cinerea*. Int. J. Mol. Sci..

[45] Jing Y., Liu J., Liu P., Ming D., Sun J. (2019). Overexpression of *TaJAZ1* increases powdery mildew resistance through promoting reactive oxygen species accumulation in bread wheat. Sci. Rep..

[46] Wu H., Ye H., Yao R., Zhang T., Xiong L. (2015). OsJAZ9 acts as a transcriptional regulator in jasmonate signaling and modulates salt stress tolerance in rice. Plant Sci..

[47] Fu J., Wu H., Ma S., Xiang D., Liu R., Xiong L. (2017). OsJAZ1 attenuates drought resistance by regulating JA and ABA signaling in rice. Front. Plant Sci..

[48] Zhu D., Cai H., Luo X., Bai X., Deyholos M.K., Chen Q., Chen C., Ji W., Zhu Y. (2012). Over-expression of a novel *JAZ* family gene from *Glycine soja*, increases salt and alkali stress tolerance. Biochem. Biophys. Res. Commun..

[49] Shi M., Zhou W., Zhang J., Huang S., Wang H., Kai G. (2016). Methyl jasmonate induction of tanshinone biosynthesis in *Salvia miltiorrhiza* hairy roots is mediated by JASMONATE ZIM-DOMAIN repressor proteins. Sci. Rep..

[50] Zheljazkov V.D., Stewart C.N., Joyce B., Baxter H., Cantrell C.L., Astatkie T., Jeliazkova E.A., Poovaiah C.R. (2018). Dual utilization of medicinal and aromatic crops as bioenergy feedstocks. J. Agric. Food Chem..

[51] Zhao D., Xu Y.W., Yang G.L., Husaini A.M., Wu W. (2013). Variation of essential oil of *Mentha haplocalyx* Briq. and *Mentha spicata* L. from China. Ind. Crops Prod..

[52] Lange B.M., Ahkami A. (2013). Metabolic engineering of plant monoterpenes, sesquiterpenes and diterpenes-current status and fu- ture opportunities. Plant Biotechnol. J..

[53] Heydari M., Zanfardino A., Taleei A., Bushehri A.A.S., Hadian J., Maresca V., Sorbo S., Napoli M.D., Varcamonti M., Basile A. (2018). Effect of heat stress on yield, monoterpene content and antibacterial activity of essential oils of *Mentha x piperita* var. Mitcham and *Mentha arvensis* var. piperascens. Molecules.

[54] Li Y., Liu Y., Ma A., Bao Y., Wang M., Sun Z. (2017). In vitro antiviral, anti-inflammatory, and antioxidant activities of the ethanol extract of *Mentha piperita* L.. Food Sci. Biotechnol..

[55] Bouvier F., Rahier A., Camara B. (2005). Biogenesis, molecular regulation and function of plant isoprenoids. Prog. Lipid Res..

[56] Sukegawa S., Shiojiri K., Higami T., Suzuki S., Arimura G.I. (2018). Pest management using mint volatiles to elicit resistance in soy: Mechanism and application potential. Plant J..

[57] Figueroa-Pérez M.G., Reynoso-Camacho R., Garcia-Ortega L.F., Guevara-González R.G. (2019). Transcriptome profiling of pep- permint (*Mentha piperita*) with improved antioxidant properties in response to salicylic acid elicitation. J. Plant Biochem. Biot..

[58] Joo-Sun S., Yung-Jin C., Yang-Do C., Soo-Un K. (1998). Role of jasmonic acid in biotransformation of (--)-isopiperitenone in suspen- sion cell culture of *Mentha piperita*. Mol. Cells.

[59] Bose S.K., Yadav R.K., Mishra S., Sangwan R.S., Singh A.K., Mishra B., Srivastava A.K., Sangwan N.S. (2013). Effect of gibber- ellic acid and calliterpenone on plant growth attributes, trichomes, essential oil biosynthesis and pathway gene expression in differential manner in *Mentha arvensis* L.. Plant Physiol. Biochem..

[60] Chrysargyris A., Papakyriakou E., Petropoulos S.A., Tzortzakis N. (2019). The combined and single effect of salinity and copper stress on growth and quality of *Mentha spicata* plants. J. Hazard. Mater..

[61] Ahkami A., Johnson S.R., Srividya N., Lange B.M. (2015). Multiple levels of regulation determine monoterpenoid essential oil compositional variation in the mint family. Mol. Plant.

[62] Reddy V.A., Wang Q., Dhar N., Kumar N., Venkatesh P.N., Rajan C., Panicker D., Sridhar V., Mao H.-Z., Sarojam R. (2017). Spearmint R2R3-MYB transcription factor MsMYB negatively regulates monoterpene production and suppresses the expression of geranyl diphosphate synthase large subunit (*MsGPPS.LSU*). Plant Biotechnol. J..

[63] Wang Q., Reddy V.A., Panicker D., Mao H.-Z., Kumar N., Rajan C., Venkatesh P.N., Chua N.H., Sarojam R. (2016). Metabolic engineering of terpene biosynthesis in plants using a trichome-specific transcription factor MsYABBY5 from spearmint (*Mentha spicata*). Plant Biotechnol. J..

[64] Tafrihi M., Imran M., Tufail T., Gondal T.A., Caruso G., Sharma S., Sharma R., Atanassova M., Atanassov L., Valere Tsouh Fokou P. (2021). The wonderful activities of the genus *Mentha*: Not only antioxidant properties. Molecules.

[65] Yu X., Liang C., Chen J., Qi X., Liu Y., Li W. (2015). The effects of salinity stress on morphological characteristics, mineral nutrient accumulation and essential oil yield and composition in *Mentha canadensis* L.. Sci. Hortic..

[66] Wang H.T., Yu X., Liu Y., Liang C.Y., Li W.L. (2013). Analysis of genetic variability and relationships among *Mentha* L. using the limonene synthase gene, *LS*. Gene.

[67] Qi X., Fang H., Yu X., Xu D., Li L., Liang C., Lu H., Li W., Chen Y., Chen Z. (2018). Transcriptome analysis of JA signal trans- duction, transcription factors, and monoterpene biosynthesis pathway in response to methyl jasmonate elicitation in *Mentha canadensis* L.. Int. J. Mol. Sci..

[68] Ku Y.S., Sintaha M., Cheung M.Y., Lam H.M. (2018). Plant hormone signaling crosstalks between biotic and abiotic stress responses. Int. J. Mol. Sci..

[69] Chen K., Li G.J., Bressan R.A., Song C.P., Zhu J.K., Zhao Y. (2020). Abscisic acid dynamics, signaling, and functions in plants. J. Integr. Plant Biol..

[70] Wang P., Yu S., Han X., Xu J., He Q., Xu S., Wang R. (2020). Identification, molecular characterization and expression of *JAZ* genes in *Lycoris aurea*. PLoS ONE.

[71] Garrido-Bigotes A., Valenzuela-Riffo F., Torrejón M., Solano R., Morales-Quintana L., Figueroa C.R. (2020). A new functional JAZ degron sequence in strawberry JAZ1 revealed by structural and interaction studies on the COI1-JA-Ile/COR-JAZs complexes. Sci. Rep..

[72] Katsir L., Schilmiller A.L., Staswick P.E., He S.Y., Howe G.A. (2008). COI1 is a critical component of a receptor for jasmonate and the bacterial virulence factor coronatine. Proc. Natl. Acad. Sci. USA.

[73] Yan C., Xie D. (2015). Jasmonate in plant defence: Sentinel or double agent?. Plant Biotechnol. J..

[74] Shen J., Zou Z., Xing H., Duan Y., Zhu X., Ma Y., Wang Y., Fang W. (2020). Genome-wide analysis reveals stress and hormone responsive patterns of *JAZ* family genes in *Camellia Sinensis*. Int. J. Mol. Sci..

[75] Grunewald W., Vanholme B., Pauwels L., Plovie E., Inzé D., Gheysen G., Goossens A. (2009). Expression of the *Arabidopsis* jasmonate signalling repressor *JAZ1/TIFY10A* is stimulated by auxin. EMBO Rep..

[76] Chung H.S., Cooke T.F., DePew C.L., Patel L.C., Ogawa N., Kobayashi Y., Howe G.A. (2010). Alternative splicing expands the repertoire of dominant JAZ repressors of jasmonate signaling. Plant J..

[77] Saha G., Park J.I., Kayum M.A., Nou I.S. (2016). A Genome-wide analysis reveals stress and hormone responsive patterns of *TIFY* family genes in *Brassica rapa*. Front. Plant Sci..

[78] Fernández-Calvo P., Chini A., Fernández-Barbero G., Chico J.M., Gimenez-Ibanez S., Geerinck J., Eeckhout D., Schweizer F., Godoy M., Franco-Zorrilla J.M. (2011). The *Arabidopsis* bHLH transcription factors MYC3 and MYC4 are targets of JAZ repressors and act additively with MYC2 in the activation of jasmonate responses. Plant Cell.

[79] Schweizer F., Fernández-Calvo P., Zander M., Diez-Diaz M., Fonseca S., Glauser G., Lewsey M.G., Ecker J.R., Solano R., Reymond P. (2013). *Arabidopsis* basic helix-loop-helix transcription factors MYC2, MYC3, and MYC4 regulate glucosinolate biosynthesis, insect performance, and feeding behavior. Plant Cell.

[80] Mao Y.B., Liu Y.Q., Chen D.Y., Chen F.Y., Fang X., Hong G.J., Wang L.J., Wang J.W., Chen X.Y. (2017). Jasmonate response decay and defense metabolite accumulation contributes to age-regulated dynamics of plant insect resistance. Nat. Commun..

[81] Song S., Qi T., Huang H., Ren Q., Wu D., Chang C., Peng W., Liu Y., Peng J., Xie D. (2011). The Jasmonate-ZIM domain proteins interact with the R2R3-MYB transcription factors MYB21 and MYB24 to affect Jasmonate-regulated stamen development in *Arabidopsis*. Plant Cell.

[82] Liu B., Seong K., Pang S., Song J., Gao H., Wang C., Zhai J., Zhang Y., Gao S., Li X. (2021). Functional specificity, diversity, and redundancy of *Arabidopsis* JAZ family repressors in jasmonate and COI1-regulated growth, development, and defense. New Phytol..

[83] Hsouna A.B., Touj N., Hammami I., Dridi K., Al-Ayed A.S., Hamdi N. (2019). Chemical composition and in vivo efficacy of the essential oil of *Mentha piperita* L. in the suppression of crown gall disease on tomato plants. J. Oleo Sci..

[84] Pang X., Feng Y.X., Qi X.J., Wang Y., Almaz B., Xi C., Du S.S. (2020). Toxicity and repellent activity of essential oil from *Mentha piperita* Linn. leaves and its major monoterpenoids against three stored product insects. Environ. Sci. Pollut. Res. Int..

[85] Liu X.J., An X.H., Liu X., Hu D.G., Wang X.F., You C.X., Hao Y.J. (2017). MdSnRK1.1 interacts with MdJAZ18 to regulate sucrose-induced anthocyanin and proanthocyanidin accumulation in apple. J. Exp. Bot..

[86] Ma Y.N., Chen M., Xu D.B., Fang G.N., Wang E.H., Gao S.Q., Xu Z.S., Li L.C., Zhang X.H., Min D.H. (2015). G-protein β subunit AGB1 positively regulates salt stress tolerance in *Arabidopsis*. J. Integr. Agr..

[87] Xu D.B., Chen M., Ma Y.N., Xu Z.S., Li L.C., Chen Y.F., Ma Y.Z. (2015). A G-protein β subunit, AGB1, negatively regulates the ABA response and drought tolerance by down-regulating AtMPK6-related pathway in *Arabidopsis*. PLoS ONE.

[88] Tian S., Liu S., Wang Y., Wang K., Yin C., Yue Y., Hu H. (2019). Genome-wide identification and characterization of JAZ protein family in two *Petunia Progenitors*. Plants.

[89] Shen Q., Zhang L., Liao Z., Wang S., Yan T., Shi P., Liu M., Fu X., Pan Q., Wang Y. (2018). The genome of *Artemisia annua* provides insight into the evolution of *Asteraceae* family and artemisinin biosynthesis. Mol. Plant.

[90] Gasteiger E., Gattiker A., Hoogland C., Ivanyi I., Appel R.D., Bairoch A. (2003). ExPASy: The proteomics server for in-depth protein knowledge and analysis. Nucleic Acids Res..

[91] Thompson J.D., Gibson T.J., Plewniak F., Jeanmougin F., Higgins D.G. (1997). The CLUSTAL_X windows interface: Flexible strat- egies for multiple sequence alignment aided by quality analysis tools. Nucleic Acids Res..

[92] Tamura K., Stecher G., Peterson D., Filipski A., Kumar S. (2013). MEGA6: Molecular evolutionary genetics analysis version 6.0. Mol. Biol. Evol..

[93] Xu D.B., Gao S.Q., Ma Y.N., Wang X.T., Feng L., Li L.C., Xu Z.S., Chen Y.F., Chen M., Ma Y.Z. (2017). The G-protein β subunit AGB1 promotes hypocotyl elongation through inhibiting transcription activation function of BBX21 in *Arabidopsis*. Mol. Plant.

[94] Livak K.J., Schmittgen T.D. (2001). Analysis of relative gene expression data using real-time quantitative PCR and the 2^−ΔΔCT^ Method. Methods.

[95] Bender C.L. (1999). Chlorosis-inducing phytotoxins produced by *Pseudomonas syringae*. Eur. J. Plant Pathol..

