# Supplementary material for: Transcriptome-Wide Identification and Characterization of the JAZ Gene Family in Mentha canadensis L
Source: Int J Mol Sci. 2021 Aug 17;22(16):8859. doi: 10.3390/ijms22168859 (PMC8396335; doi:10.3390/ijms22168859)
Supplement: Supplementary file 1 [file ijms-22-08859-s001.zip › ijms-1329270-supplementary.pdf]

Supplementary Materials

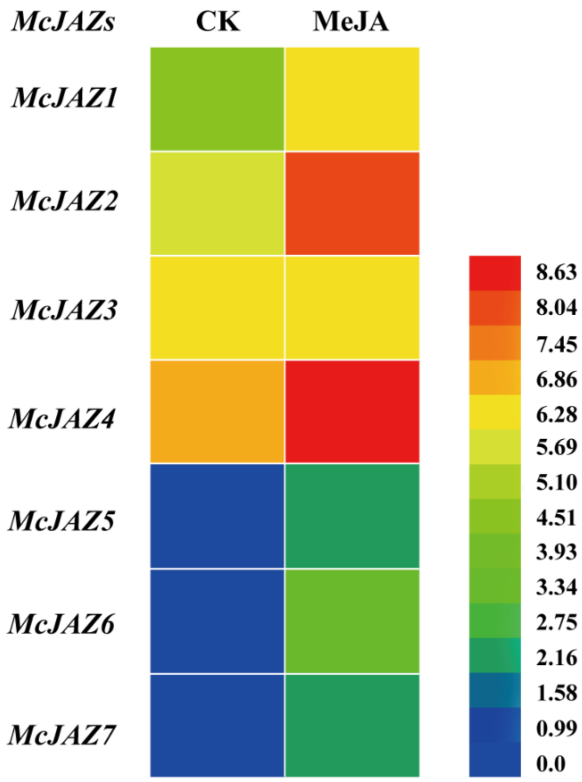

Supplemental Figure S1. Expression heatmaps of *McJAZ* genes under normal condition and MeJA treatment condition.

Heatmap analysis of *McJAZ* genes of *M. canadensis* under normal condition (CK) and MeJA treatment condition. The data are from our previous published transcriptome data (SRP132644, Qi *et al*., 2018). The color scale at the right represents the value of log2-transformed reads per kilobase per million mapped reads.

Supplemental Table S1. Pairwise amino acid sequence identity of different McJAZ proteins in *M. canadensis*.

Proteins	McJAZ1	McJAZ2	McJAZ3	McJAZ4	McJAZ5	McJAZ6	McJAZ7
McJAZ1	***	27.80	17.92	14.18	20.99	16.18	16.60
McJAZ2		***	14.45	21.37	25.51	16.18	10.98
McJAZ3			***	25.65	12.43	9.25	6.94
McJAZ4				***	15.99	10.31	7.63
McJAZ5					***	18.62	18.72
McJAZ6						***	80.80
McJAZ7							***

Supplemental Table S2. Primers used in this study.

**Name**	**Primer sequence (5'-3')**
**Primers used for *JAZ* genes clone**	
McJAZ1-F	*TTGCAAAGCCTCTCTCTC*
McJAZ1-R	*ATCTAAAAGGCAAGAAGAGG*
McJAZ2-F	*AATATTCTCGCAACTTTTCGTC*
McJAZ2-R	*TGGAGTTTGAGACGCTGTG*
McJAZ3-F	*GTTCCTGAGAAAAAGAAGAAGTG*
McJAZ3-R	*GCATCTTCTGCTAAAGTCAGC*
McJAZ4-F	*GAGATTTCATGGGGTTGAAC*
McJAZ4-R	*CCCTATTAGAAACAAAGTTGACAC*
McJAZ5-F	*CAGACGTGAAATTCTCAGC*
McJAZ5-R	*GGCACAAATAATTTCTTCTCG*
McJAZ6-F	*CTCTCTCTAGATTCATCTATCTTG*
McJAZ6-R	*GACGTAGCTAGTGTCTAGTAATG*
McJAZ7-F	*CTCATTAGATTCATCTCTTGATC*
McJAZ7-R	*CCTTCCCAAAATATCTAATC*
**Primers used for quantitative real-time (qRT)-PCR**	
qRT-McJAZ1-F	*GTCTCCAGCTCGAGAAAAGG*
qRT-McJAZ1-R	*CTCCCTTCCAAGAAGAGCCT*
qRT-McJAZ2-F	*GTTCCGAAAACGTCCAGC*
qRT-McJAZ2-R	*GGAACACTGGAGGCCGAC*
qRT-McJAZ3-F	*TACAGCTCCAGTTCAGGCAG*
qRT-McJAZ3-R	*CGTTCGTTTCAGCCCTCA*
qRT-McJAZ4-F	*ACATGCAAGCACCTGCCT*
qRT-McJAZ4-R	*GCAGATGCAGCAACGGA*

qRT-McJAZ5-F	*CTCACTTGCCGGTTTTCG*
qRT-McJAZ5-R	*TTGATATGGGCAAATCTCCAC*
qRT-McJAZ6-F	*CGCTAACCAGTCCCACTCC*
qRT-McJAZ6-R	*GTACACCGGCGAATTCATC*
qRT-McJAZ7-F	*GCAACTGCAATTTGGAGCT*
qRT-McJAZ7-R	*TCATCGACATGCCAGTCG*
β-actin-F	*CCAGGAATTGCTGATAGGATGAG*
β-actin-R	*GCGCCACCACCTTAATCTTC*
**Primers used to generate DNA constructs for the yeast two-hybrid assay**	
BD-McJAZ1-F	*GGAGGACCTGCATATGATGTCTAGTTCAAGAATGT*
BD-McJAZ1-R	*GGATCCCCGGGAATTCTTATAATCTGAGCTCAAGC*
BD-McJAZ2-F	*GGAGGACCTGCATATGATGGCTTCCTTCGAGATCGTC*
BD-McJAZ2-R	*GGATCCCCGGGAATTCCTACCTGTCTCGTCTCTTCTCC*
BD-McJAZ3-F	*GGAGGACCTGCATATGATGGAGAGAGATTTCATGG*
BD-McJAZ3-R	*GGATCCCCGGGAATTCTTAATTTGCTGCTGGAACT*
BD-McJAZ4-F	*GGAGGACCTGCATATGATGGGGTTGAACGCTAAG*
BD-McJAZ4-R	*GGATCCCCGGGAATTCTCAGCACACACATTGAGT*
BD-McJAZ5-F	*GGAGGACCTGCATATGATGGCCAAATCTTCATCTC*
BD-McJAZ5-R	*GGATCCCCGGGAATTCCTAATATGGGGACACCGT*
BD-McJAZ6-F	*GGAGGACCTGCATATGATGAAACGCAACTGCAACT*
BD-McJAZ6-R	*GGATCCCCGGGAATTCCTAGTGTCTAGTAATGGAG*
BD-McJAZ7-F	*GGAGGACCTGCATATGATGAAACGCAACTGCAATTT*
BD-McJAZ7-R	*GGATCCCCGGGAATTCCTACCGTCTAGTAATCGG*
BD-AtCOI1-F	*GGAGGACCTGCATATGATGGAGGATCCTGATATCAAGAG*
BD-AtCOI1-R	*GGATCCCCGGGAATTCTCATATTGGCTCCTTCAGGACT*
AD-McJAZ1-F	*AGATTACGCTCATATGATGTCTAGTTCAAGAATGT*
AD-McJAZ1-R	*TCATCTGCAGCTCGAGCTTATAATCTGAGCTCAAGC*
AD-McJAZ2-F	*AGATTACGCTCATATGATGGCTTCCTTCGAGATCGTC*
AD-McJAZ2-R	*TCATCTGCAGCTCGAGCCTACCTGTCTCGTCTCTTCTCC*
AD-McJAZ3-F	*AGATTACGCTCATATGATGGAGAGAGATTTCATGG*
AD-McJAZ3-R	*TCATCTGCAGCTCGAGCTTAATTTGCTGCTGGAACT*
AD-McJAZ4-F	*AGATTACGCTCATATGATGGGGTTGAACGCTAAG*
AD-McJAZ4-R	*TCATCTGCAGCTCGAGCTCAGCACACACATTGAGT*
AD-McJAZ5-F	*AGATTACGCTCATATGATGGCCAAATCTTCATCTC*
AD-McJAZ5-R	*TCATCTGCAGCTCGAGCCTAATATGGGGACACCGT*
AD-McJAZ6-F	*AGATTACGCTCATATGATGAAACGCAACTGCAACT*
AD-McJAZ6-R	*TCATCTGCAGCTCGAGCCTAGTGTCTAGTAATGGAG*
AD-McJAZ7-F	*AGATTACGCTCATATGATGAAACGCAACTGCAATTT*
AD-McJAZ7-R	*TCATCTGCAGCTCGAGCCTACCGTCTAGTAATCGG*
**Primers used to generate DNA constructs for protein localization**	
McJAZ1-GFP-F	*GGGGCCCGGGGTCGACATGTCTAGTTCAAGAATGT*
McJAZ1-GFP-R	*TACCGGATCCACTAGTTAATCTGAGCTCAAGC*
McJAZ2-GFP-F	*GGGGCCCGGGGTCGACATGGCTTCCTTTGAGAACGTC*

McJAZ2-GFP-R	*TACCGGATCCACTAGTCCTGTCTCGTCTCTTCTCC*
McJAZ3-GFP-F	*GGGGCCCGGGGTCGACATGGAGAGAGATTTCATGG*
McJAZ3-GFP-R	*TACCGGATCCACTAGT ATTTGCTGCTGGAACT*
McJAZ4-GFP-F	*GGGGCCCGGGGTCGACATGGGGTTGAACGCTAAG*
McJAZ4-GFP-R	*TACCGGATCCACTAGTGCACACACATTGAGT*
McJAZ5-GFP-F	*GGGGCCCGGGGTCGACATGGCCAAATCTTCATCTC*
McJAZ5-GFP-R	*TACCGGATCCACTAGTATATGGGGACACCGT*
McJAZ6-GFP-F	*GGGGCCCGGGGTCGACATGAAACGCAACTGCAACT*
McJAZ6-GFP-R	*TACCGGATCCACTAGTGTGTCTAGTAATGGAG*
McJAZ7-GFP-F	*GGGGCCCGGGGTCGACATGAAACGCAACTGCAATTT*
McJAZ7-GFP-R	*TACCGGATCCACTAGTCCGTCTAGTAATCGG*
